# The early diversification of ray‐finned fishes (Actinopterygii): hypotheses, challenges and future prospects

**DOI:** 10.1111/brv.12907

**Published:** 2022-10-03

**Authors:** Struan Henderson, Emma M. Dunne, Sophie A. Fasey, Sam Giles

**Affiliations:** ^1^ School of Geography, Earth and Environmental Sciences University of Birmingham Edgbaston Birmingham B15 2TT UK; ^2^ GeoZentrum Nordbayern Friedrich‐Alexander Universität Erlangen‐Nürnberg (FAU) Loewenichstraße 28 Erlangen 91054 Germany; ^3^ Department of Earth Sciences Natural History Museum Cromwell Road London SW7 5BD UK

**Keywords:** fossils, ichthyology, diversity, Palaeozoic, Actinopterygii, sampling biases

## Abstract

Actinopterygii makes up half of living vertebrate diversity, and study of fossil members during their Palaeozoic rise to dominance has a long history of descriptive work. Although research interest into Palaeozoic actinopterygians has increased in recent years, broader patterns of diversity and diversity dynamics remain critically understudied. Past studies have investigated macroevolutionary trends in Palaeozoic actinopterygians in a piecemeal fashion, variably using existing compendia of vertebrates or literature‐based searches. Here, we present a comprehensive occurrence‐based dataset of actinopterygians spanning the whole of the Palaeozoic. We use this to produce the first through‐Palaeozoic trends in genus and species counts for Actinopterygii. Diversity through time generally tracks metrics for sampling, while major taxonomic problems pervading the Palaeozoic actinopterygian record obscure diversity trends. Many described species are concentrated in several particularly problematic ‘waste‐basket’ genera, hiding considerable morphological and taxonomic diversity. This taxonomic confusion also feeds into a limited understanding of phylogenetic relationships. A heavy sampling bias towards Europe and North America exists in both occurrence databases and available phylogenetic matrices, with other regions underrepresented despite yielding important data. Scrutiny of the extent to which spatial biases influence the actinopterygian record is lacking, as is research on other forms of bias. Low richness in some time periods may be linked to geological biases, while the effects of taphonomic biases on Palaeozoic actinopterygians have not yet been investigated. Efforts are already underway both to redescribe poorly defined taxa and to describe taxa from underrepresented regions, helping to address taxonomic issues and accuracy of occurrence data. New methods of sampling standardisation utilising up‐to‐date occurrence databases will be critical in teasing apart biological changes in diversity and those resulting from bias. Lastly, continued phylogenetic work will enable the use of phylogenetic comparative methods to elucidate the origins of actinopterygian biogeography and subsequent patterns of radiation throughout their rise to dominate aquatic faunas.

## I. INTRODUCTION

Reconstructions of deep‐time biodiversity patterns are critical to understanding the evolution of life on Earth. However, deciphering whether these patterns represent true changes in biodiversity is a key challenge for palaeobiologists (Raup, 1972, 1976; Sepkoski, 1981; Alroy *et al*., 2008). The past 20 years have seen rapid growth in the number of quantitative studies employing fossil occurrence data to estimate patterns of diversity in vertebrate groups. The majority of work on vertebrate diversity through time focuses on either individual taxonomic groups of tetrapods (e.g. Alroy, 2009; Benson *et al*., 2010; Mannion *et al*., 2011, 2019; Butler *et al*., 2011; Butler, Benson & Barrett, 2013; Brocklehurst, Kammerer & Fröbisch, 2013; Pearson *et al*., 2013; Cleary *et al*., 2015, 2018, 2020; Bennett *et al*., 2018; Cantalapiedra, Domingo & Domingo, 2018; Cantalapiedra *et al*., 2021; Brown *et al*., 2019; Driscoll *et al*., 2019; Celis *et al*., 2020) and fishes (Sallan & Coates, 2010; Koot, 2013; Lloyd & Friedman, 2013; Sansom, Randle & Donoghue, 2015; Romano *et al*., 2016), or large‐scale analyses of all tetrapods using publicly available, community‐led databases such as the Paleobiology Database (PBDB; paleobiodb.org) (Sahney, Benton & Ferry, 2010; Close *et al*., 2017, 2019, 2020*a*; Dunne *et al*., 2018; Dunne, 2020). These studies have allowed insight into evolutionary dynamics in deep time, the assembly of ancient and modern ecosystems, and revealed major changes in diversification, extinction, and paleoecology. For example, studies of Palaeozoic vertebrates have illuminated the rise of jawed vertebrates from the Silurian to the Devonian (Sansom *et al*., 2015), a major shift from placoderm‐ and sarcopterygian‐dominated faunas to chondrichthyan‐ and actinopterygian‐dominated faunas after the end‐Devonian mass extinction (Sallan & Coates, 2010), and changes in Palaeozoic tetrapod diversity in relation to palaeoenvironments (Dunne *et al*., 2018; Pardo *et al*., 2019).

Despite accounting for roughly half of extant vertebrate species (Nelson, Grande & Wilson, 2016), research on the diversity of actinopterygians over long evolutionary timescales comprises only a fraction of macroevolutionary studies. Ray‐finned fishes likely evolved in the Silurian (Zhu *et al*., 2009) with the crown group originating close to the Devonian–Carboniferous boundary (Giles *et al*., 2017), but diversity dynamics throughout the Palaeozoic are poorly understood due to the limited number of studies utilising occurrence‐based datasets. This reflects a broader palaeontological trend of understudy into the fossil record of fishes (Friedman & Sallan, 2012). Notable exceptions include diversity and faunal analyses of Middle Devonian to Mississippian gnathostomes (Sallan & Coates, 2010); an analysis of British fish richness (Lloyd & Friedman, 2013); and a study on Permo‐Triassic osteichthyans (Romano *et al*., 2016). Some studies have used compendia of first and last appearances to plot counts through time (Benton, 1993; Patterson, 1994; Sepkoski, 2002; Blieck, 2011; Friedman & Sallan, 2012). Other works examine patterns of biodiversity across long periods of time using publicly available occurrence data (e.g. PBDB), although they present aggregated data for numerous groups of ‘fishes’, or an even broader set of taxa such as nektonic metazoans (e.g. Whalen & Briggs, 2018; Harper, Cascales‐Miñana & Servais, 2020; Close *et al*., 2020*b*).

While these studies represent an important first foray into understanding Palaeozoic actinopterygian evolution, there have been limited syntheses that take the accuracy of the ray‐fin fossil record into account, which is a major barrier to reconstructing long‐term evolutionary patterns. Previous attempts either focus on the UK and include non‐actinopterygian fishes (Lloyd & Friedman, 2013), do not cover the entire Palaeozoic (Sallan & Coates, 2010; Romano *et al*., 2016), or are broader in scope without as much focus on the suitability of data and barriers to interpreting diversity patterns (Sallan, 2014). Friedman & Sallan (2012) note the lack of such investigation for fishes, and, through a qualitative survey, suggest that geological and taxonomic biases likely impact studies of the diversity of fishes through time. Here, we summarise the current state of research on the Palaeozoic fossil record of actinopterygians and present a new occurrence database spanning the Palaeozoic in an attempt to answer the following questions: (*i*) how has our understanding of the Palaeozoic actinopterygian fossil record changed over time; (*ii*) what are the trends in face‐value diversity through the Palaeozoic; (*iii*) how do sampling and other biases affect our understanding of Palaeozoic actinopterygian diversity through time; and (*iv*) how do taxonomic problems and existing phylogenetic analyses hinder our interpretation of the Palaeozoic actinopterygian fossil record?

## II. CURRENT HYPOTHESES OF PALAEOZOIC ACTINOPTERYGIAN DIVERSITY

### (1) Past studies

**Fig. 1:**
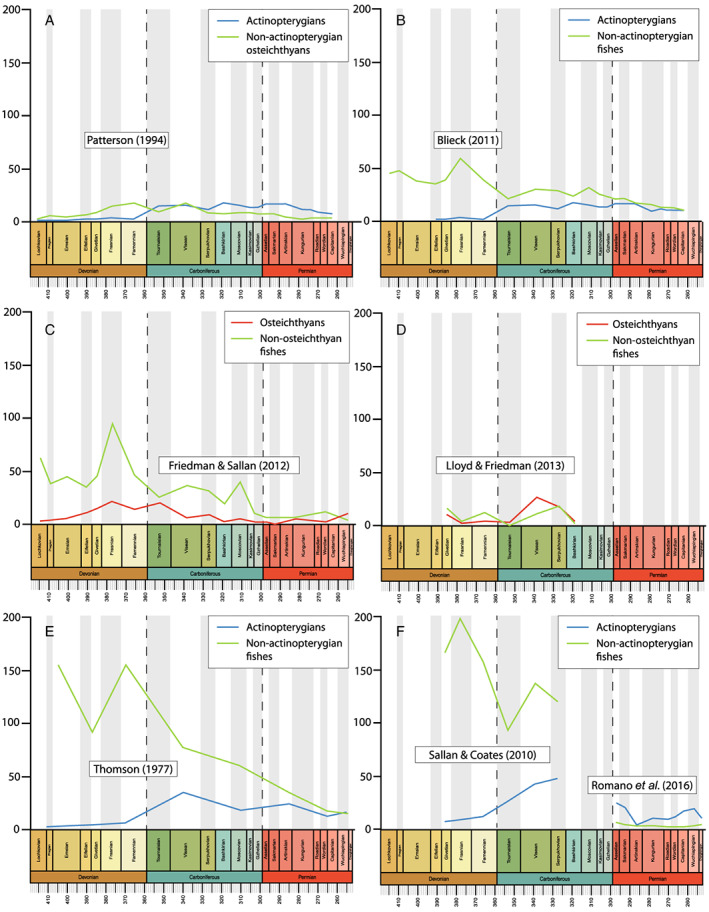
Diversity of Palaeozoic fishes through time presented in previous studies. (A) Family‐level diversity curves of actinopterygians and non‐actinopterygian osteichthyans (Patterson, 1994; using data from Benton, 1993). (B) Family‐level diversity curves of actinopterygians and non‐actinopterygian fishes (Blieck, 2011; using data from Benton, 1993). (C) Genus‐level diversity of marine osteichthyans and non‐osteichthyan fishes, excluding conodonts (Friedman & Sallan, 2012; using data from Sepkoski, 2002). (D) Genus‐level diversity of British osteichthyans and non‐osteichthyan fishes (Lloyd & Friedman, 2013). (E) Genus‐level diversity of actinopterygians and non‐actinopterygian fishes (Thomson, 1977; using data from Romer, 1966). (F) Genus‐level diversity of actinopterygians and non‐actinopterygian fishes (Sallan & Coates, 2010; Romano *et al*., 2016).

Although our understanding of patterns of actinopterygian diversity lags behind that of other groups, a number of studies over the past few decades have investigated fish diversity at different taxonomic levels and geological scales (Fig. 1). Initially, these approaches used published compendia to generate family‐ and/or genus‐level diversity curves. The first major attempt (Thomson, 1977) used data from Romer's (1966) compendium to plot genus‐ and family‐level diversity of Phanerozoic ‘fishes’ (Acanthodii, Agnatha, Chondrichthyes, Chondrostei, Holostei, Placodermi, Sarcopterygii and Teleostei; Fig. 1E). In subsequent years, several studies used family‐level data from Benton (1993) to investigate osteichthyan diversity through the Palaeozoic. Patterson (1994) plotted diversity curves for osteichthyans as well as stem‐actinopterygians, stem‐neopterygians and stem‐teleosts, encompassing all Palaeozoic actinopterygians included in the parent dataset (Fig. 1A). Blieck (2011; Fig. 1B) and Benton (2014: fig. 2.11) also used data compiled by Benton (1993) to plot family‐level diversity curves of vertebrates from the Ordovician to Triassic, though did not focus on actinopterygians. Additionally, Friedman & Sallan (2012) used an existing marine dataset (Sepkoski, 2002) to present genus‐level diversity patterns of all ‘fishes’ (vertebrates excluding Tetrapoda and including Conodonta) throughout the Phanerozoic (Fig. 1C).

Other attempts have used literature‐based datasets to interrogate patterns of diversity. Sallan & Coates (2010) assembled a dataset of gnathostome occurrences from 66 localities spanning the Middle Devonian (Givetian) to early Carboniferous (Serpukhovian) and presented diversity curves of gnathostomes (Acanthodii, Actinopterygii, Chondrichthyes, Placodermi, Sarcopterygii, Tetrapoda; Fig. 1F). Lloyd & Friedman (2013) compiled data from a variety of sources, both as a means of comparing datasets (Agassiz, 1833; Carroll, 1988; Benton, 1993; Sepkoski, 2002; Palaeobiology Database, downloaded on 31/05/12) and to investigate the diversity of Phanerozoic ‘fishes’ (excluding Conodonta) with a particular focus on the fossil record of Great Britain (Fig. 1D). Romano *et al*. (2016) and Vázquez & Clapham (2017) compiled datasets that commence in the Asselian (early Permian) and encompass osteichthyans (Actinistia, Dipnoi, Holostei, ‘Palaeopterygii’, ‘Subholostei’ and Teleosteomorpha: Romano *et al*., 2016; Fig. 1F) and marine fishes [Osteichthyes (excluding Dipnoi) and Chondrichthyes (excluding Acanthodii); Vázquez & Clapham, 2017].

**Fig. 2:**
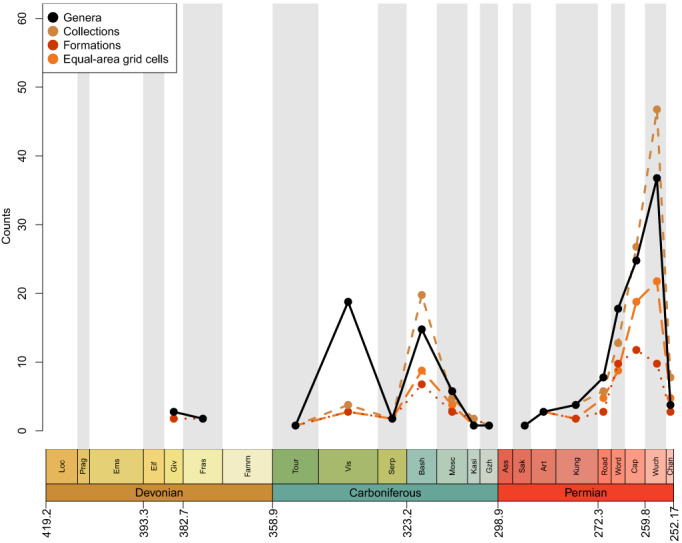
Raw counts of Palaeozoic actinopterygian genera (black, solid line), collections (brown, short‐dashed line), formations (red, dotted line) and equal‐area grid cells (orange, long‐dashed line) entered in the Paleobiology Database (PBDB).

As in Lloyd & Friedman (2013), a large proportion of recent diversity studies for fossil groups utilise occurrence data from the PBDB, a public resource that is voluntarily maintained by an international group of palaeontologists. However, most diversity studies on actinopterygians have relied on published compendia or datasets compiled directly from the literature, i.e. without use of the PBDB [see Vázquez & Clapham (2017) for an exception]. There have not yet been enough efforts to enter occurrence data for osteichthyans, and particularly actinopterygians, into the PBDB to represent their record accurately, as discussed by Lloyd & Friedman (2013) and evidenced by current PBDB Palaeozoic actinopterygian diversity curves for genera, collections, formations and equal‐area grid cells (Fig. 2). Several periods throughout the Devonian and early Permian lack entries entirely, and no time period contains more than 50 occurrences: the average number of occurrences is less than eight per interval, while the median is three. Diversity levels appear to fluctuate wildly during the Carboniferous before a precipitous rise through the Permian and steep drop in the Changhsingian. The PBDB data presented here (Fig. 2) is intended as a snapshot of the currently available occurrence data, highlighting that research effort to contribute Palaeozoic actinopterygian occurrences to the database has thus far been minimal. Improving this record represents a priority for future studies, and efforts are currently underway to expand the actinopterygian PBDB record.

The studies introduced above differ greatly in their sampling and spread of taxa, but collectively they provide an indication of the general patterns of changes in actinopterygian diversity through time, as summarised below and in Figs 1 and 2.

### (2) Devonian diversity patterns

All studies covering the Devonian depict very low counts of actinopterygians (Thomson, 1977: fig. 7; Patterson, 1994: fig. 1; Sallan & Coates, 2010: fig. 1; Blieck, 2011: fig. 2) (Fig. 1), and PBDB occurrences are lacking (Fig. 2). Thomson (1977), Patterson (1994) and Sallan & Coates (2010) show a gradual rise from the Middle to Late Devonian. Blieck (2011), however, figures a small peak in the Frasnian, likely due to the Gogo and Gladbach faunas (Sallan & Coates, 2010), while only a handful of genera from the Givetian and Frasnian (and none in the Famennian) have been entered into the PBDB. Only four genera (eight species) of actinopterygians are entered in the PBDB for the entire Devonian; fewer than the number described in the literature for just the Famennian (Dunkle, 1964; Dunkle & Schaeffer, 1973; Taverne, 1997; Daeschler, 2000; Prokofiev, 2002; Friedman & Blom, 2006).

While new taxa are still being described, actinopterygians appear to be genuinely rare in Devonian deposits, especially relative to other taxa (Friedman, 2015: fig. 4). Reclassification of *Meemannia* Zhu *et al*. 2004 as a ray‐finned fish rather than a lobe‐finned fish (Lu *et al*., 2016) filled a conspicuous temporal gap in early actinopterygian evolution, but this taxon remains the only actinopterygian known amongst roughly 20 species from this locality. Choo *et al*. (2019) recently described a new genus of actinopterygian from the highly diverse Frasnian Gogo Formation, which is known primarily for its placoderm and sarcopterygian faunas. Although ray fins account for only five species out of around 50 Gogo taxa (Long & Trinajstic, 2010, 2017; Sallan & Coates, 2010: fig. 2) they comprise a large proportion of specimens, indicating faunal abundance despite taxonomic paucity. Even more recently, Newman *et al*. (2021) described a new species of *Cheirolepis* Agassiz 1835 from the Givetian of Svalbard, found alongside roughly 20 non‐actinopterygian fishes. Similarly, a new site from the Famennian of Belgium has yielded microremains of an undescribed actinopterygian, amidst large numbers of other vertebrates (Olive *et al*., 2015*a*,*b*, 2016, 2020).

Recent work on historically undersampled regions has revealed numerous new taxa, although overall taxonomic diversity of actinopterygians remains relatively low throughout the Devonian. Isolated jaw elements, body impressions and scales from Famennian deposits in South Africa likely represent a single actinopterygian amid a diverse array of other fishes (Gess & Whitfield, 2020), while renewed prospecting in the contemporary Maïder Basin of Morocco has produced remains of a single articulated actinopterygian (Frey *et al*., 2018) amongst its well‐known placoderm and chondrichthyan assemblages. New South American discoveries include evidence of a stegotrachelid actinopterygian from the Frasnian of Colombia (Olive *et al*., 2019), the first actinopterygian remains from the Devonian of the Parnaíba Basin of Brazil (Pais de Rezende *et al*., 2021), and a new circumpolar species from the Middle Devonian (Figueroa, Weinschütz & Friedman, 2021). As in other localities, non‐actinopterygian fishes dominate these faunas (Janvier, 2007; Janvier & Maisey, 2010; Figueroa & Machado, 2018). The low diversity of actinopterygians also correlates with their limited morphological disparity, contrasting with the vast array of anatomies, and presumably ecologies, exhibited by Devonian sarcopterygians and placoderms (Anderson *et al*., 2011). While important for understanding the early evolution of the group, these scattered reports of new Devonian taxa are unlikely to change existing overarching hypotheses of actinopterygian diversity: as minor faunal components represented by a small number of taxa relative to other fish groups.

### (3) Carboniferous diversity patterns

**Fig. 3:**
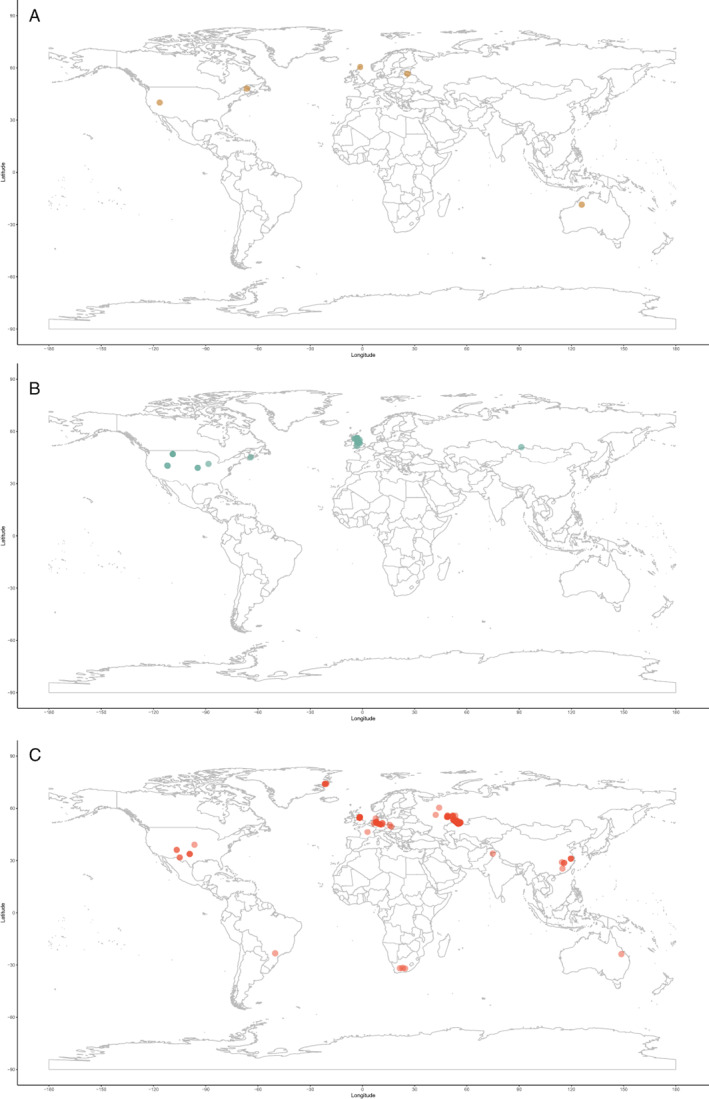
Geographic spread of actinopterygian occurrences entered in the Paleobiology Database (PBDB) for the (A) Devonian; (B) Carboniferous; and (C) Permian.

Previous diversity studies consistently report a large increase in actinopterygian taxonomic diversity in the earliest Carboniferous following the end‐Devonian mass extinction (EDME). This increase is somewhat reflected in the data currently entered in the PBDB, although entries are extremely limited both taxonomically (only 51 taxa from 92 localities are entered) and geographically (all but one of the entries are from USA and UK localities; Fig. 3B). Thomson's (1977) counts of ‘chondrostean’ genera (which encompasses all Devonian and Carboniferous actinopterygians) rise sharply in the Mississippian, as does Patterson's (1994) stem‐actinopteran family‐level count. Sallan & Coates (2010) show this significant change in absolute and relative diversity most clearly in their presentation of faunal composition from the Devonian into the Carboniferous (Sallan & Coates, 2010: fig. 2; see also Friedman, 2015: fig. 4). This sharp rise is especially notable because the early Carboniferous (Tournaisian and early Visean) coincides with ‘Romer's Gap’, an apparent hiatus in the fossil record of tetrapods (and other animals) variably explained as either a period of poor sampling (Romer, 1956), low atmospheric oxygen (Ward *et al*., 2006) or recovery following the EDME (Sallan & Coates, 2010). Recent concerted efforts have begun to populate Romer's Gap, indicating that poor sampling accounted for most of the apparent paucity of the record (Clack *et al*., 2019; Otoo *et al*., 2019). The diversification of actinopterygians immediately following the EDME likely represents an adaptive radiation seeded by very few – or perhaps just one – actinopterygian lineages (Sallan & Friedman, 2012; Sallan, 2014; Giles *et al*., 2017), although this hypothesis has not been explicitly tested. The contrast between diverse (e.g. in Russia; Alekseev *et al*., 1994) and depleted (e.g. in Morocco; Frey *et al*., 2018) early Tournaisian faunas exemplifies the uncertainty of the relative contributions of extinction recovery, poor sampling and spatial bias to the observed fossil record, although potential differences between local environmental conditions are an important consideration.

Raw genus diversity increases into the Visean from Tournaisian levels in most previous analyses (Patterson, 1994; Sallan & Coates, 2010; Blieck, 2011) and the PBDB (Fig. 2). The fossil record of Great Britain exhibits a particularly extreme increase in osteichthyan richness, most likely due to the very richly sampled Visean deposits of Scotland (Dineley & Metcalf, 1999). This rise coincides with a proliferation of new morphologies and ecologies, likely *via* multiple independent acquisitions of key traits such as durophagy and deep‐ and eel‐like‐bodies (Sallan & Friedman, 2012; Sallan, 2012, 2014; Sallan & Coates, 2013; Friedman, 2015; Friedman *et al*., 2018). This gradual rise in richness, accompanied by morphological and functional diversification, may represent a classic extinction recovery and adaptive radiation (Sallan & Friedman, 2012; Sallan, 2014).

Previous studies suggest conflicting patterns of actinopterygian raw diversity into the Serpukhovian. Patterson (1994) and Blieck (2011) report a decrease in family counts, in contrast to a slight increase in genus counts in Sallan & Coates (2010). The diversity curve of Thomson (1977) only separates data into Mississippian and Pennsylvanian bins, and therefore lacks the temporal resolution to allow comparison. Discrepancy between the trends in Sallan & Coates (2010), Patterson (1994) and Blieck (2011) may be due to poor higher‐level taxonomy in actinopterygians. For example, the highly diverse Bear Gulch fauna likely drives the rise in actinopterygian diversity in Sallan & Coates (2010), but this is not captured in higher‐level family counts due to the aggregation of genera in broad, ill‐defined families. Additionally, the Serpukhovian decrease in PBDB occurrence data contrasts with other studies (e.g. Sallan & Coates, 2010) and is, in part, due to inconsistencies between regional substages and International Commission on Stratigraphy (ICS) stages. For example, in the PBDB, Bear Gulch localities are included in the Bashkirian due to the age range of the Arnsbergian, but more accurately should be placed in the Serpukhovian.

It is difficult to reconstruct patterns of diversity in the late Carboniferous due to a lack of compiled occurrence data across the Pennsylvanian. Sallan & Coates' (2010) range ends at the Mississippian, while Romano *et al*.’s (2016) data begin in the Asselian. Thomson's (1977) genus counts decrease from the Mississippian to the Pennsylvanian, however family counts of actinopterygians increase from the Serpukhovian to the Bashkirian (Patterson, 1994; Blieck, 2011). For the Moscovian–Gzhelian the only data for actinopterygians are the family counts derived from Benton (1993); these show gradual decreases from the Bashkirian to the Moscovian, and again from the Moscovian to plateau in the Kasimovian and Gzhelian (Patterson, 1994; Blieck, 2011). Importantly, counts of families remain at roughly the same level as they were in the Tournaisian and Visean. Counts of osteichthyan genera are not discernible for this period in Friedman & Sallan (2012: fig. 2), and there are no Kasimovian or Gzhelian occurrences in the British fossil record (Lloyd & Friedman, 2013). Counts of genera in the PBDB decrease throughout the Pennsylvanian (Fig. 2), although this also appears to be a result of low data entry: Pennsylvanian PBDB actinopterygian occurrences derive from important localities for other groups [e.g. Linton for early tetrapods (Hook & Baird, 1986); Mazon Creek for arthropods (Clements, Purnell & Gabbott, 2019)].

Reported overall trends in actinopterygian diversity in the Carboniferous are consequently unclear. Genus‐level counts are suggestive of a gradual rise throughout the Mississippian (Sallan & Coates, 2010), with a subsequent drop in the Pennsylvanian (Thomson, 1977). This contrasts with family counts, which are relatively stable except for minor deviations in the Serpukhovian and Bashkirian.

### (4) Permian diversity patterns

Genus‐ and family‐level counts in previous studies agree on the general trend of actinopterygian diversity in the Permian, although differ at finer timescales. The highest counts are observed in the early Permian in curves derived from Benton's (1993) dataset (Patterson, 1994; Blieck, 2011) and Thomson's (1977) genus‐level data. Occurrence‐based datasets also show a peak in the early Permian, although limited to the Asselian and Sakmarian, likely driven by freshwater Lagerstätte (Romano *et al*., 2016). However, very few early Permian occurrences of actinopterygians have been entered into the PBDB (Fig. 2), although the geographic spread of occurrences in the Permian PBDB is substantially greater than the Devonian or Carboniferous (Fig. 3C). Genus‐ and family‐level trends deviate from one another in the Artinskian: the family curve stays more or less stable, whereas genus richness decreases substantially. Family‐level counts drop in the Kungurian and remain roughly at this level, with minor fluctuations, until the end‐Permian. Genus richness in Thomson's (1977) curves for ‘chondrosteans’ drop in the middle Permian and rise slightly in the late Permian; the late Permian also sees the first appearance of holosteans. Counts in the finer‐scale dataset of Romano *et al*. (2016) rise gradually from the Roadian–Wuchiapingian, reaching close to early Permian levels before dropping in the Changhsingian. Unlike the early Permian, PBDB data closely reflect the trends of Romano *et al*. (2016) in large part due to targeted entry of marine fishes for studies relating to the End‐Permian Mass Extinction (e.g. by Vázquez & Clapham, 2017). It is clear, however, that the substantial freshwater actinopterygian fossil record from the late Carboniferous‐early Permian (Beltan, 1978, 1981; Forey & Young, 1985; Murray, 2000; Soler‐Gijón & Moratalla, 2001; Evans, 2005; Štamberg & Zajíc, 2008; Šimůnek & Cleal, 2020) has not yet been entered into the PBDB.

While previous studies have established a broad understanding of general diversity trends in the Palaeozoic, patterns differ depending on the taxonomic level and geological scale investigated, and there has not yet been a through‐Palaeozoic study focussing solely on actinopterygians. At present, publicly available occurrence databases lack the level of detail required for reconstructing long‐term diversity through the Palaeozoic, necessitating the collation of occurrences spanning the Palaeozoic.

## III. MATERIALS AND METHODS

### (1) Data preparation

Global occurrences of Palaeozoic Actinopterygii were compiled from the published literature. Taxonomically indeterminate occurrences (i.e. those that could not be confidently assigned to a valid genus or species) were excluded, as were occurrences represented solely by scales or teeth (i.e. only body fossils were retained). The cleaned dataset comprises 1611 occurrences, representing 468 species belonging to 225 genera, from 507 unique geographic locations. We recognise that databases compiled from the published literature are subject to bias (Alroy, 2010*a*,*b*,*c*; Clapham *et al*., 2016; Close *et al*., 2018), however collating and examining occurrences present in the literature provides a foundation upon which to build. The database includes taxon identity, locality name, locality coordinates, stratigraphy, region and country, age (ICS stage and regional substage), authority naming the taxon and the year the species was described. These data are available as online supporting information (Table S1) and are in the process of being uploaded to the PBDB.

### (2) Occurrence data

The length of stratigraphic stages drastically differs within the Palaeozoic. For example, the Kasimovian is 3.3 million years (Myr) in length, compared to the 15.8 Myr long Visean. As the length of intervals may impact richness trends (Raup, 1972; Smith & McGowan, 2011), occurrence data were placed in composite intervals of roughly equal length (~9 Myr) intervals following Close *et al*. (2017, 2020*a*,*b*), as well as in standard stratigraphic stages (Lochkovian–Changhsingian). In order to form equal length intervals, some stratigraphic stages were combined (e.g. the Kasimovian and Gzhelian) and others were split (e.g. the Visean) (Table S2). Interval ranges were updated to reflect most recent stage boundaries according to the ICS (Cohen, Harper & Gibbard, 2021). Equal‐length intervals were compared with standard stratigraphic stages to obtain an indication of the effect of interval length on diversity counts.

Face‐value (=raw, uncorrected, or observed) genus and species richness at ‘global’ scales are presented with the proviso that face‐value diversity counts may be highly misleading. While ‘global’ curves likely represent the extent of spatial sampling rather than global palaeodiversity (Close *et al*., 2017, 2020*a*,*b*), face‐value richness counts allow for comparison with previous diversity curves (e.g. Thomson, 1977; Sallan & Coates, 2010; Romano *et al*., 2016) and for an initial exploration of gross Palaeozoic actinopterygian diversity. ‘Global’ (gamma scale) face‐value richness curves were computed using sampled‐in‐bin counts of occurrences. Counts of geographic localities and geological units (unique formations, members, groups, etc.) were used to provide an indication of sampling effort. Occupied equal‐area grid cells, i.e. the number of 50 km^2^ cells on a global map (constructed using the *dggridR* R package; Barnes, 2021) that contained unique localities, were calculated as a further measure of sampling. Localities were plotted on a modern world map to show the scope of present‐day sampling.

Linear regressions were conducted to investigate the relationship between counts of taxa and various sampling metrics (localities, formations and equal‐area grid cells), as well as with sea level through time (data from Hannisdal & Peters, 2011). All analyses were conducted within R 4.1.0 (R Core Team, 2020).

### (3) Collector's curves

We extracted taxonomic identity, country, authority naming the taxon and the year the species was described from the occurrence database, resulting in a total of 516 species. Collector's curves showing cumulative counts of the total number of species described through time globally and within the UK were then plotted.

## IV. RESULTS

### (1) Occurrence data

**Fig. 4:**
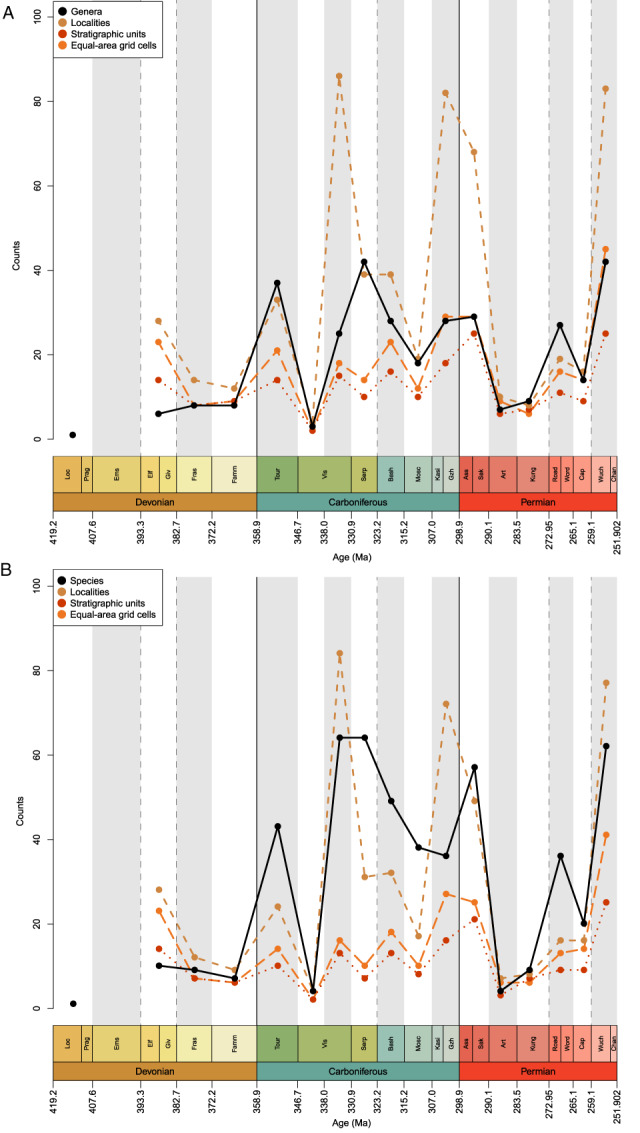
Raw counts of Palaeozoic actinopterygian (A) genera and (B) species (black, solid line) in roughly equal‐length intervals (see Table S2). Collections (brown, short‐dashed line), formations (red, dotted line) and equal‐area grid cells (orange, long‐dashed line) are also plotted.

Overall diversity trends in the equal length genus‐level, raw occurrence dataset are hard to discern (Fig. 4A), but genus richness is highest during the late Permian (Lopingian; Wuchiapingian) and mid‐Carboniferous (Serpukhovian). The lowest levels are seen in the Devonian: only a single taxon is identified in the Lochkovian (Lu *et al*., 2016), and, aside from contentious scale‐based taxa, no ray‐finned fish are known from Pragian or Emsian deposits. Richness increases marginally from the Eifelian and Givetian to the Frasnian, remaining flat in the Famennian. Counts increase substantially from the Famennian to the Tournaisian, before decreasing dramatically in the early Visean (Chadian‐Holkerian) and rising in the late Visean (Asbian–Brigantian). A peak in the Serpukhovian is followed by a decrease in counts in the Bashkirian and Moscovian and another modest rise across the Carboniferous‐Permian boundary. A large decrease in the Artinskian sees raw genus counts return to late Devonian levels. Counts increase again from the Artinskian trough to the Wordian, followed by a fall in the Capitanian and final peak in the Lopingian.

Raw species richness broadly follows the same pattern, though with some notable departures (Fig. 4B). The highest species counts are in the late Visean (Asbian–Brigantian) and Serpukhovian, although are only marginally lower in the Lopingian (Wuchiapingian and Changsinghian) and earliest Permian (Asselian and Sakmarian). In contrast to patterns of genus richness, species richness decreases slightly from the Middle to the Late Devonian. Furthermore, Kasimovian and Gzhelian species richness is lower than the Moscovian, meaning that, unlike in the raw genus counts, richness noticeably increases across the Carboniferous to Permian boundary.

**Fig. 5:**
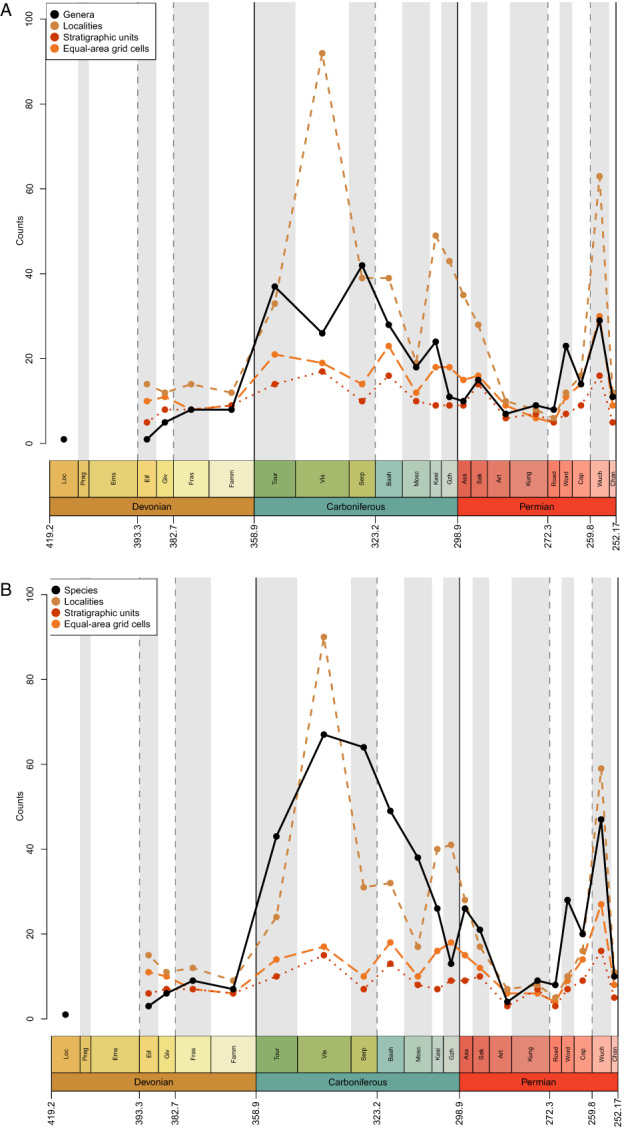
Raw counts of Palaeozoic actinopterygian (A) genera and (B) species (black, solid line) in standard International Commission on Stratigraphy (ICS) stages. Collections (brown, short‐dashed line), formations (red, dotted line) and equal‐area grid cells (orange, long‐dashed line) are also plotted.

Comparison of counts of taxa in roughly equal‐length stages with counts in ICS stages shows that the choice of sampling interval strongly influences richness trends (Figs 4 and 5). Devonian trends for genus and species richness are similar (Fig. 5A), however trends in the early Carboniferous and Permian differ, with ICS stage counts instead largely resembling (as expected) previous studies analysing these periods (e.g. Sallan & Coates, 2010; Romano *et al*., 2016). Genus richness is highest in the Serpukhovian, however a notable departure from previous hypotheses is the Tournaisian peak in genus richness followed by a drop in the Visean. This results from a discrepancy in the age of the diverse Waaipoort Formation: previous work counted these deposits as Visean (Sallan & Coates, 2010), suggesting a gradual rise in richness through the Mississippian, while recent studies shift the age back to the Tournaisian (Lakin *et al*., 2016), in turn altering Mississippian richness trends. There is then a general decline throughout the Pennsylvanian that reaches a trough in the final interval of the Carboniferous, the Gzhelian (Fig. 5A). Richness then decreases in the Asselian and rises in the Sakmarian before a drop in the Artinskian (the lowest count of genera throughout the Carboniferous and Permian). Genus richness fluctuates through the Kungurian (increase from Artinskian), Roadian (decrease from Kungurian), Wordian (increase from Roadian), Capitanian (decrease from Wordian), Wuchiapingian (increase from Capitanian) and Changhsingian (decrease from Wuchiapingian); these trends are broadly similar to Romano *et al*. (2016). Species richness trends are generally the same (albeit exaggerated) as those of genus richness, excepting the Visean, which becomes the most speciose interval of the Palaeozoic (Fig. 5B).

Face‐value richness at both genus and species level closely tracks proxies for sampling effort; where the number of localities, formations and equal‐area grid cells are high, richness is also high (Fig. 4). Notable exceptions to this trend are the Eifelian–Givetian and Serpukhovian. Discrepancy in the Eifelian–Givetian is due to the widespread occurrence of *Cheirolepis* at a time when actinopterygians had very low relative diversity. In the Serpukhovian, high genus counts despite a decrease in sampling metrics is a result of the diverse Bear Gulch fauna (Lund, Greenfest‐Allen & Grogan, 2012). In addition, three large peaks in counts of localities in the late Visean, Kasimovian and Gzhelian and Asselian and Sakmarian are a result of intense sampling of localised regions with homogenous contemporary faunas [e.g. Midland Valley of Scotland, Visean (Dineley & Metcalf, 1999); Boskovice Graben, late Carboniferous and early Permian (Štamberg, 2007; Štamberg & Zajíc, 2008)], and thus do not correspond with peaks in genus richness. However, there are species‐level richness counts peaks in the late Visean and Asselian and Sakmarian (Fig. 4B).

First inspection reveals multiple sources of bias in the Palaeozoic actinopterygian fossil record. The clear differences between the species‐ and genus‐level curves highlight issues with problematic ‘waste‐basket’ genera containing vast numbers of species, while overall face‐value richness appears to track sampling metrics. Regressions (Fig. S1) show that genus richness positively correlates with number of localities (*R* ^2^ = 0.47, *p* = 0.003, Fig. S1A), stratigraphic units (*R* ^2^ = 0.34, *p* = 0.019, Fig. S1B) and occupied equal‐area grid cells (*R* ^2^ = 0.39, *p* = 0.009, Fig. S1C). Removing the highly diverse Bear Gulch and Glencartholm assemblages strengthens these relationships. As expected, interval length does not correlate with richness when using roughly equal‐length intervals (*R* ^2^ = 0.16, *p* = 0.128, Fig. S1D). Overall genus richness also significantly correlates with sea level (*R* ^2^ = 0.47, *p* = 0.003, Fig. S1E). This relationship persists when analysing isolated counts of freshwater genera (*R* ^2^ = 0.44, *p* = 0.005, Fig. S1F), although counts of marine genera do not significantly correlate with sea level (*R* ^2^ = 0.15, *p* = 0.135, Fig. S1G). This significant correlation, with both overall genera and freshwater genera, disappears when the Devonian stages are removed (*R* ^2^ = 0.14, *p* = 0.205, Fig. S1H; *R* ^2^ = 0.20, *p* = 0.129, Fig. S1I).

### (2) Distribution of Palaeozoic actinopterygians

**Fig. 6:**
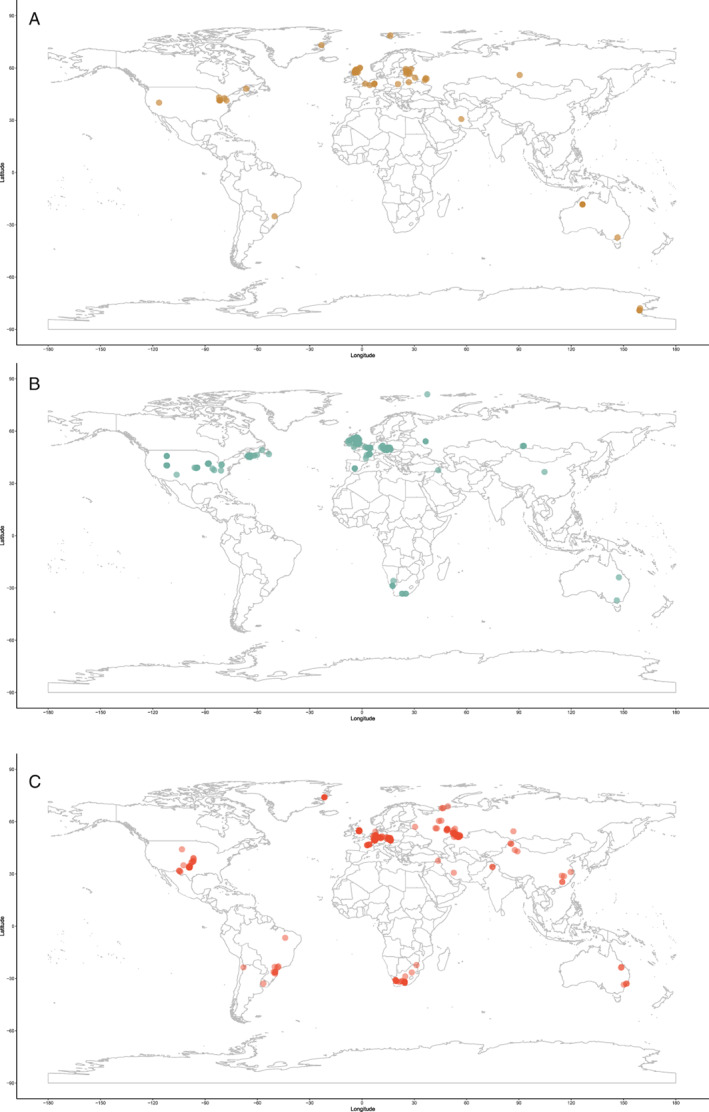
Geographic spread of actinopterygian occurrences in the (A) Devonian; (B) Carboniferous; and (C) Permian.

A global map of occurrences gives a broad overview of the distribution of actinopterygian localities through the Devonian, Carboniferous and Permian (Fig. 6), showing that published occurrences are overwhelmingly located in Europe and North America. Here we break down the global data in order to (*a*) understand better the distributions of actinopterygians through the Palaeozoic and (*b*) identify widespread taxa or regions that share taxonomic affinities.

#### (a) United Kingdom and the Republic of Ireland

Eifelian and Givetian occurrences in the well‐known Orcadian basin precede a nearly continuous Carboniferous record of actinopterygians in the British Isles, which persists until the end of the Moscovian. This is followed by a total lack of occurrences until the extensively sampled Wuchiapingian deposits of the Raisby and Marl Slate Formations (Westoll, 1934, 1941*b*).

**Fig. 7:**
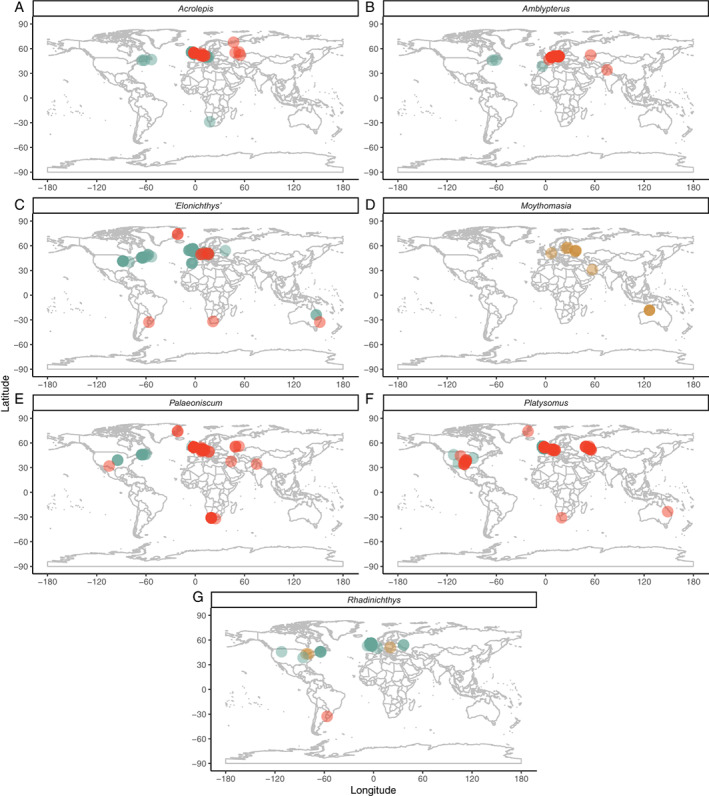
Distribution of the most speciose and widespread actinopterygian genera, with occurrences coloured according to the ICS colours for the period in which they occur (Devonian – brown; Carboniferous – green; Permian – red): (A) *Acrolepis*; (B) *Amblypterus*; (C) ‘*Elonichthys*’; (D) *Moythomasia*; (E) *Palaeoniscum*; (F) *Platysomus*; and (G) *Rhadinichthys*.

The earliest Devonian taxon from this region, *Cheirolepis* from the Eifelian of Scotland (Pearson & Westoll, 1979), is also present in North America (Arratia & Cloutier, 1996, 2004), the Baltic (Mark‐Kurik, 2000) and Spitsbergen (Newman *et al*., 2021), while *Stegotrachelus* (Givetian; Swartz, 2009) may also occur in central Europe (Ørvig, 1960). Some Tournaisian actinopterygians in the British Isles are present at other isolated Northern Hemisphere localities, for example in the Tournaisian of Russia (Lebedev, 1996) and Serpukhovian of the USA (Lowney, 1980). However, by far the most common genera throughout the Carboniferous are ‘*Elonichthys*’ and *Rhadinichthys*, which are also geographically widespread (Fig. 7C, G). While there are some endemic genera that are locally widespread and present at many localities [e.g. *Eurynotus*, *Nematoptychius* (Traquair, 1908; Moy‐Thomas & Dyne, 1938; Friedman *et al*., 2018)], the other most common Carboniferous occurrences in British and Irish deposits are of globally distributed genera (*Platysomus*, *Palaoniscum*, *Acrolepis*, Fig. 7A, E, F). The late Permian fish fauna (Marl Slate and Raisby Formations) is very similar to that of contemporary German deposits (Kupferschiefer and Zechstein Formations; Westoll, 1941*a*).

#### (b) Western Europe

In Western Europe, isolated occurrences in the Frasnian, Famennian, Tournaisian, Visean, Serpukhovian and Bashkirian precede more considerable numbers of occurrences in the Kasimovian, Asselian and Sakmarian (Fig. 6). These late Carboniferous and early Permian Western European localities yield taxa that are shared with contemporary central European deposits, including *Paramblypterus*, ‘*Elonichthys*’, *Progyrolepis*, *Bourbonnella* and *Aeduella* (Štamberg, 2006; Štamberg & Zajíc, 2008). Of these genera, *Bourbonnella* and *Progyrolepis* are also present in the USA (Dunkle, 1946; Dalquest & Kocurko, 1988; Mickle, 2011), while numerous other genera found in Western Europe are also widespread: *Cheirodus* (Bashkirian, France; Derycke, Cloutier & Candilier, 1995) also occurs in the UK (Visean–Bashkirian; Traquair, 1890); *Gonatodus* (Visean, Belgium) in the UK (Gardiner, 1967) and USA (Hannibal, 2020); *Mesonichthys* (Serpukhovian, France; Derycke *et al*., 1995) in the UK (Bashkirian; Elliott, 2016) and Uruguay (early Permian; Beltan, 1978); *Pygopterus* (Bashkirian, Belgium; Derycke *et al*., 1995) in Germany, Greenland and the UK [all Wuchiapingian (King, 1850; Woodward, 1891; Aldinger, 1937; Holzapfel & Malzahn, 1984; Diedrich, 2009; Hosgör & Štamberg, 2014)]; and *Rhadinichthys* (Serpukhovian, Belgium; Derycke *et al*., 1995) is present across the globe (Fig. 7G).

#### (c) Central Europe

Devonian occurrences in Germany and Poland (Givetian–Frasnian) represent the earliest in Central Europe, with a subsequent gap encompassing the entirety of the early Carboniferous. Actinopterygians later occur in the Moscovian, Kasimovian and Gzhelian, with particularly large numbers of occurrences in the latter two stages (largely in Czechia, with a few occurrences in the Gzhelian of Germany (Štamberg & Zajíc, 2008; Schindler, 2018*a*)]. The early Permian of Czechia and Germany are also extensively sampled. Isolated Artinskian and Kungurian occurrences precede a hiatus until the considerable counts in the Wuchiapingian of Germany, stemming from the famous Kupferschiefer and Zechstein Formations. In total these deposits contribute a reasonable number of genera to the global count.

Two Devonian genera (*Moythomasia*, *Rhadinichthys*) are present at numerous localities globally (Fig. 7D, G), although the third, *Stegotrachelus*, is only present in the Givetian of Scotland (Swartz, 2009). ‘*Elonichthys*’, *Palaeoniscum*, *Amblypterus* and *Acrolepis*, all of which have notably global distributions (Fig. 7A–C, E), comprise a large number of occurrences in Central Europe. Intense sampling of Central European deposits has resulted in abundant occurrences of locally widespread taxa. Many of these taxa are endemic to the region [*Spinarichthys*, *Rhabdolepis*, *Zaborichthys* (Štamberg, 1991, 2016*a*; Štamberg & Zajíc, 2008; Schindler, 2018*b*)], and others occur at isolated localities outside Central Europe (e.g. *Sphaerolepis*, USA; Olson, 1967) or in the broader palaeogeographic region encompassing present‐day Europe and North America (e.g. *Aeduella*, *Bourbonnella*, *Paramblypterus*, *Progyrolepis*, *Pygopterus*).

#### (d) Eastern Europe

Actinopterygians are reported from the Eifelian, Givetian, Frasnian Famennian and Tournaisian of Eastern Europe and European Russia. Occurrences are absent from the remainder of the Carboniferous, and only a single Kungurian occurrence is known. In stark contrast, the middle and late Permian of Russia are heavily sampled, with numerous occurrences in the Roadian, Wordian, Capitanian and Wuchiapingian.

Devonian occurrences yield the globally distributed *Cheirolepis* (Mark‐Kurik, 2000) and *Moythomasia* (Fig. 7D; Sallan & Coates, 2010) for the most part, with only a single endemic genus, *Krasnoyarichthys* (Prokofiev, 2002). Widespread genera are also present in the Tournaisian [e.g. ‘*Elonichthys*’, *Rhadinichthys*: Fig. 7C, G (Alekseev *et al*., 1994; Yankevich & Minikh, 1998; Golubev, 2001; Minikh & Minikh, 2009; Minikh, Minikh & Yankevich, 2016)], albeit alongside a notable number of unique genera [e.g. *Oxypteriscus*, *Ministrella*, *Palaeobergia* (Berg, 1958; Matveeva, 1958)]. A small number of genera are in common with the Tournaisian of the UK (*Aetheretmon*, *Strepheoschema*; Lebedev, 1996) and Siberian Russia (*Ganolepis*, *Grassator*; Lebedev, 1996).

Similarly, the middle Permian occurrences range on a spectrum from endemic to widespread genera. *Platysomus* has the highest number of occurrences (Fig. 7F), and *Acrolepis* and *Palaeoniscum* (Fig. 7A, E; Golubev, 2001; Tverdokhlebov *et al*., 2005; Minikh & Minikh, 2009; Nurgaliev, Silantiev & Nikolaeva, 2015; Bakaev & Kogan, 2020) are also common. *Varialepis* and *Alilepis* are present in contemporary middle Permian deposits from the USA (Ivanov, Nestell & Nestell, 2013; Ivanov *et al*., 2020) while other frequently occurring genera are endemic and span multiple stages (e.g. *Toyemia*, *Isadia*, *Geryonichthys*, *Kargalichthys*; Tverdokhlebov *et al*., 2005).

#### (e) North America

Relatively few actinopterygians occur in the Devonian of North America, with limited occurrences in the Givetian, Frasnian and Famennian. Tournaisian occurrences predominantly derive from Canada [comprising an assortment of globally widespread genera such as *Acrolepis*, ‘*Elonichthys*’, and *Rhadinichthys*: Fig. 7A, C, G (Rygel *et al*., 2006; Mickle, 2017)]. Visean localities are rare. By contrast, the Serpukhovian is highly diverse, although all but one of the Serpukhovian occurrences are from Bear Gulch deposits (Weems & Windolph, 1986). There are occurrences throughout the Pennsylvanian, however the majority occur in the Moscovian [Mazon and Yellow Creek localities (Newberry, 1856; Schultze & Bardack, 1987)].

Occurrences are limited throughout the early and middle Permian, mirroring the overall Palaeozoic actinopterygian record. There are no late Permian occurrences in continental North America, although Wuchiapingian deposits containing actinopterygians are present in Greenland (Aldinger, 1937). ‘*Elonichthys*’, *Platysomus* and *Palaeoniscum* comprise three of the four most abundantly occurring genera in North America. In addition, the collective occurrences of these genera in North America range from the earliest Carboniferous to the end‐Permian proving them not only geographically (Fig. 7C, E, F) but temporally widespread. However, *Rhadinichthys* and *Acrolepis* are more restricted temporally in North America than their other global occurrences (Fig. 7A, G). The overwhelming majority of Bear Gulch taxa – the source of most of North American actinopterygian diversity – are endemic to the locality, although a small number of genera are present elsewhere [e.g. *Mesopoma* and *Phanerosteon* (Traquair, 1881; White, 1927; Moy‐Thomas & Dyne, 1938; Moy‐Thomas, 1938; Gardiner, 1985)]. More broadly, actinopterygian genera from North America are also present in numerous European deposits: *Alilepis* (Russia; Minikh *et al*., 2016), *Bourbonnella* [Czechia, France, Spain (Heyler, 1977; Soler‐Gijón & Moratalla, 2001; Štamberg, 2007)], *Parahaplolepis* (UK; Elliott, 2014, 2016), *Progyrolepis* [Czechia, Spain, France (Forey & Young, 1985; Heyler, 2000; Soler‐Gijón & Moratalla, 2001; Štamberg & Zajíc, 2008)], *Pyritocephalus* [Czechia, UK (Štamberg, 1991; Elliott, 2014)], *Sphaerolepis* (Czechia; Štamberg & Zajíc, 2008) and *Varialepis* (Russia; Nurgaliev *et al*., 2015).

#### ( f ) Africa

African occurrences predominantly derive from South African deposits, with a scattering of contributions from Namibia and Zimbabwe. These sparse occurrences are separated by large temporal gaps: actinopterygians are only reported from the Tournaisian, Gzhelian (or Asselian, age is uncertain; Murray, 2000), Artinskian, Capitanian and Changhsingian (Fig. 6). The diverse Tournaisian fauna of the Waaipoort Formation is endemic, with no taxa found at any other Palaeozoic locality (Gardiner, 1969; Evans, 2005). Indeed, the majority of African taxa are endemic [Gzhelian‐Changhsingian, e.g. *Namaichthys* (Gürich, 1923; Murray, 2000); Changhsingian, e.g. *Bethesdaichthys* and *Kompasia* (Bender, 2001, 2004)], although they are occasionally accompanied by widespread genera such as *Palaeoniscum* [Changhsingian, *P. bainii* (Egerton, 1856); Artinskian, *P. capensis* (Murray, 2000; Evans, 2005)], ‘*Elonichthys*’ (*E. whaitsi*; Jubb & Gardiner, 1975), *Acrolepis* (Gzhelian or Asselian, *A*. sp.; Murray, 2000) and *Platysomus* (Artinskian, *P*. sp.; Evans, 2005) (Fig. 7A, C, E, F). *Watsonichthys* (a genus present in Visean and Serpukhovian deposits of Scotland) is also reported in the Gzhelian (or Asselian) and Artinskian of southern Africa (Jubb & Gardiner, 1975; Evans, 2005).

#### (g) Asia

Despite the earliest actinopterygian occurring in the Devonian (Lochkovian) of China (Lu *et al*., 2016), actinopterygians subsequently only occur in China in the Bashkirian (Lu, 2002) and late Permian (Wang *et al*., 2007). Other central and eastern Asian occurrences partially populate this gap: Famennian and Tournaisian occurrences are present in Siberia, while deposits from eastern Kazakhstan (most notably those of the Kalyn‐Kara; Kazantseva‐Selezneva, 1980, 1981) yield actinopterygians from the late Carboniferous and early Permian. Some indeterminate actinopterygians also occur in the Devonian of South East Asia (Wang, Qu & Zhu, 2010). Together, these occurrences constitute a low proportion of global counts of genera. None of the taxa present in these regions, with one exception [*Saurichthys*, found in both late Permian Chinese and Russian deposits (Liu & Wei, 1988; Tverdokhlebov *et al*., 2005; Minikh & Minikh, 2009)], are found in any other Palaeozoic locality or time stage.

By contrast, occurrences in the Famennian, Tournaisian and Capitanian of the Middle East [Iran and Turkey (Hampe *et al*., 2013; Hosgör & Štamberg, 2014)] and Middle Permian of South Asia (India; Bandyopadhyay, 1999) yield a small number of genera (*Amblypterus*, *Canobius*, *Moythomasia*, *Palaeoniscum*, *Pygopterus*, and *Rhadinichthys*), all of which are found in numerous other regions of the world (Fig. 7).

#### (h) South America

Until recently, taxonomically determinate occurrences of Palaeozoic actinopterygians in South America were restricted to the Permian. The recent discovery of an actinopterygian from the Middle Devonian of Brazil (Figueroa *et al*., 2021) extends the record back some 83 million years (excluding indeterminate late Devonian occurrences; Arratia & Cione, 1996), resulting in a substantial occurrence gap. South American actinopterygians occur throughout the Permian, yet apart from the diverse Rio Negro (San Gregorio Formation) fauna from Uruguay (Beltan, 1978), these occurrences stem from a few disparate, isolated localities, and produce comparatively low numbers of genera.

The vast majority of South America taxa are endemic to the region, with some notable exceptions. The Rio Negro fauna contains species of two very common genera – *Rhadinichthys* (*R. rioniger*) and ‘*Elonichthys*’ (‘*E*.’ *macropercularis*) [Fig. 7C, G (Beltan, 1978; Cione *et al*., 2010)] – as well as less‐common genera that are nonetheless also present in more fully sampled regions. *Mesonichthys* (*M. antipodeus* from Rio Negro) is also present in the Carboniferous (Serpukhovian–Moscovian) of Belgium and the UK (Derycke *et al*., 1995; Elliott, 2016), and *Coccocephalichthys* (*C. tesselatus* from Rio Negro) is present in both the UK (Bashkirian; Poplin & Véran, 1996) and USA (Gzhelian; Poplin, 1974).

#### (i) Oceania

The only occurrences from Oceania are from Australia, where actinopterygians are present in the Devonian [Givetian and Frasnian (Long, 1988; Long & Trinajstic, 2010)] and early Carboniferous [Tournaisian and Visean (Long, 1988; Holland *et al*., 2006)], followed by a ~65 Myr gap until the mid‐late Permian [Capitanian, Wuchiapingian and Changhsingian (Woodward, 1931; Campbell & Phuoc, 1983)]. Relatively few genera comprise these occurrences, the majority of which are endemic (e.g. *Mimipiscis*, *Ebenaqua*, *Mansfieldiscus*), although there are also occurrences of the widespread genera *Moythomasia* (*M. durgaringa*) and ‘*Elonichthys*’ (‘*E*.’ *davidi*) (Fig. 7C, D). Notably, the Frasnian is the most diverse stage due to the Gogo Formation localities, which yield nearly as many genera as the remainder of the Palaeozoic occurrences.

### (3) Collector's curves

**Fig. 8:**
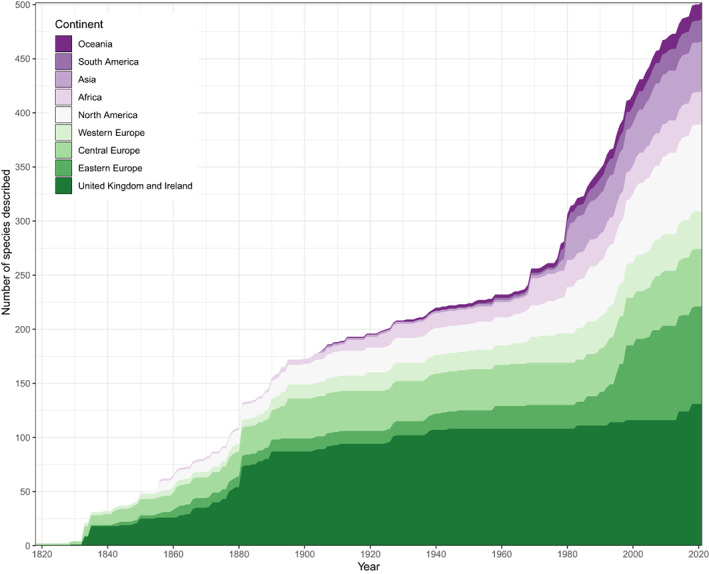
Collector's curve of the global Palaeozoic actinopterygian fossil record, divided by present‐day geographic region.

We compiled collector's curves for Palaeozoic actinopterygians to examine whether the asymptote observed by Lloyd & Friedman (2013) for the British fossil fish record is upheld when restricted to Palaeozoic actinopterygians and is extended beyond Great Britain. An asymptote is observed when considering Palaeozoic actinopterygians from the British Isles (Fig. 8). The number of described taxa starts to plateau in the late 19th century, largely due to the foundational monographic descriptions of Agassiz (1833) and Traquair (1877*b*). A slight increase in recent years indicates a resurgence of interest focussed around computed tomography (CT)‐based redescriptions and taxonomic splitting of classic taxa held in museums (e.g. Coates & Tietjen, 2018), as well as new collection and local taxonomic reviews (e.g. Elliott, 2014, 2016). While unlikely to alter large‐scale diversity patterns (Lloyd & Friedman, 2013) this uptick is suggestive of further hidden diversity in the fossil record of Palaeozoic actinopterygians in Great Britain, particularly with regard to redescription of material that has been untouched since the 19th and early 20th century.

The global collector's curve, however, presents a very different trend (Fig. 8). During the 19th century, the global curve roughly tracks that of the British curve, albeit with slightly higher cumulative counts. This is in part due to the works of Agassiz (1833) and Traquair (1877*a*,*b*), who also described actinopterygians from Belgium, France and Germany, with other important contributions from the USA and Europe (e.g. Newberry, 1856; Hancock & Atthey, 1872; Frič, 1879). Throughout the 20th century, however, the global curve departs from the British curve, continuing to rise steadily. Part of this can be traced to significant contributions from Aldinger (1937) and Gardiner (1969), who described new taxa from Greenland and South Africa, respectively. From the late 1960s the global collector's curve accelerates at a faster and steadier rate than at any time previously, corroborating statements that the fossil record of Palaeozoic actinopterygians is undersampled (Sallan & Coates, 2010).

This accelerated rate of descriptions is a combined result of a steady description of isolated occurrences (e.g. Daeschler, 2000; Prokofiev, 2002; Friedman & Blom, 2006; Long, Choo & Young, 2008; Mickle & Bader, 2009; Mickle, 2011, 2017, 2018; Choo, 2015; Giles *et al*., 2015; Figueroa *et al*., 2021; Newman *et al*., 2021; Štamberg & Steyer, 2021) as well as descriptions of new, diverse, highly productive fish faunas such as Bear Gulch (Lowney, 1980; Lund & Poplin, 1997; Lund, 2000; Poplin & Lund, 2000; Mickle, Lund & Grogan, 2009; Grogan & Lund, 2015), Mazon Creek (Schultze & Bardack, 1987), the Waaipoort Formation (Gardiner, 1969), Rio Negro (Beltan, 1989), Kalyn‐Kara (Kazantseva‐Selezneva, 1981) and the Gogo Formation (Gardiner & Bartram, 1977; Choo, Long & Trinajstic, 2009; Choo *et al*., 2019; Choo, 2012).

A geographical breakdown of the regions yielding new actinopterygian genera and localities reveals that Europe and North America are the most intensely sampled regions in the Palaeozoic actinopterygian fossil record. New European fishes account for the largest increase in descriptions in the last three decades, while contributions from North America are also increasing, although the mechanisms differ between Europe and North America. For example, increased sampling of multiple localities has contributed to the rising rate of new descriptions from Europe. Extensive work in Central Europe (Štamberg, 2007, 2010, 2013, 2016*a*,*b*, 2021; Štamberg & Zajíc, 2008; Choo, 2015) and European Russia (Minikh, 1992, 1998; Esin, 1995; Yankevich & Minikh, 1998; Minikh & Minikh, 2009; Minikh *et al*., 2016; Bakaev & Kogan, 2020) in recent years is responsible for the increase from these regions (Fig. 8). Actinopterygians are also being described from new British (Elliott, 2016) and Western European (Giles *et al*., 2015; Štamberg & Steyer, 2021) deposits. By contrast, the majority of new species from North America stem from focussed efforts on well‐known localities, such as Bear Gulch (Lowney, 1980; Lund & Melton, 1982; Lund & Poplin, 1997, 1999; Lund, 2000; Poplin & Lund, 2000, 2002; Mickle *et al*., 2009; Grogan & Lund, 2015), Kinney Brick Quarry (Gottfried, 1987, 1992; Bardack, 1992; Zidek, 1992; Williams & Lucas, 2013; Stack *et al*., 2021), and Mazon Creek (Bardack, 1979; Schultze & Bardack, 1987). Comparatively few taxa derive from more depauperate localities (Mickle, 2017, 2018; Wilson, Pardo & Anderson, 2018).

While historically understudied regions are producing new taxa, sampling and descriptions from well‐sampled regions are still outpacing them. The relative proportion of descriptions from African deposits has decreased, as except for a handful of taxa from the Beaufort Group (e.g. Bender, 2001, 2002, 2004, 2005), no taxa have been described since the mid‐20th century (Gardiner, 1969). New fishes from Siberia (Kazantseva‐Selezneva, 1980) and Kazakhstan (Kazantseva‐Selezneva, 1981) boost counts of taxa from Asia in the late‐20th century, with new descriptions published steadily in subsequent years (Poplin *et al*., 1991; Prokofiev, 2002, 2005; Zhu *et al*., 2006; Wang *et al*., 2007). Descriptions from South America follow a similar pattern, with an early burst (Beltan, 1978) followed by irregular but sustained contributions (Malabarba, 1988; Beltan, 1989; Cox & Hutchinson, 1991; Richter & Breitkreuz, 1997; Martha, 2002; Figueiredo & Carvalho, 2004; Hamel, 2005; Dias, 2012; Figueroa *et al*., 2021), notably from the Paraná Basin of Brazil (Cox & Hutchinson, 1991; Figueiredo & Carvalho, 2004; Hamel, 2005; Dias, 2012). Descriptions from Oceania [comprised exclusively of Australian fishes (Woodward, 1931, 1940; Gardiner & Bartram, 1977; Campbell & Phuoc, 1983; Long, 1988; Choo *et al*., 2009, 2019; Choo, 2012)] consistently comprise a very small proportion of the global collector's curve.

New actinopterygian taxa are being erected both from newly discovered deposits and through revisiting and revising existing museum collections. It is likely that further diversity is hiding in the extensive collections of museums in historically well‐sampled regions (e.g. Natural History Museum, London; American Museum of Natural History, New York City) – Mickle (2017) notes hundreds of specimens of three early Carboniferous genera in North American museums. Notably, museum collections will be crucial in augmenting occurrence data, as they likely contain vastly more unique localities than are recorded in the primary literature (by over an order of magnitude; Marshall *et al*., 2018). In parallel, sampling of new localities in underrepresented regions is likely to yield new taxa as the sampling universe expands (Raup, 1972; Close *et al*., 2018). The widespread adoption of CT scanning will continue to facilitate valuable redescriptions and taxonomic revisions of such material and remains an important avenue for research (Giles & Friedman, 2014; Giles *et al*., 2015, 2017; Pradel *et al*., 2016; Coates & Tietjen, 2018; Friedman *et al*., 2018; Argyriou *et al*., 2018; Figueroa, Friedman & Gallo, 2019). Both new exploration and revisiting museum collections will be important in increasing our understanding of Palaeozoic actinopterygians.

## V. CHALLENGES TO DECIPHERING THE PALAEOZOIC ACTINOPTERYGIAN FOSSIL RECORD ACCURATELY

### (1) Fossil record biases

A major obstacle to interpreting the evolution of Palaeozoic actinopterygians accurately is the various forms of biases that pervade their fossil record, which are related to geological, geographic, and anthropogenic factors. Investigations into the effect of geological, spatial and taphonomic biases on the actinopterygian fossil record are in their infancy, and the extent to which observed patterns of diversity are driven by biases is far from understood. Previous studies posit that changes in richness of the fossil fish record through time likely represent changes in sampling (Friedman & Sallan, 2012). Furthermore, the number of occupied grid cells has been suggested as the best proxy for explaining the richness of all fishes in the fossil record of Great Britain, although osteichthyan richness does not correlate with any proxy (Lloyd & Friedman, 2013). Actinopterygian richness in the Palaeozoic, however, tracks sampling proxies such as localities, formations and equal‐area grid cells (Fig. 4). A common suggestion in the literature is that the late Palaeozoic record is poorly sampled, particularly in terms of marine deposits, and that this leads to low levels of diversity (Hurley *et al*., 2007; Near *et al*., 2012; Broughton *et al*., 2013). Freshwater occurrences of actinopterygians dominate much of the Permian (Romano *et al*., 2016; Smithwick & Stubbs, 2018) and some of this skew away from marine deposits may have been linked to the formation of Pangaea and coincident reductions in coastline (Friedman & Sallan, 2012). Pinpointing the extent to which geological, spatial and taphonomic biases drive the actinopterygian record is a critical next step in understanding the evolution of actinopterygians in the Palaeozoic.

#### (a) Geological biases

The extent to which observed patterns of diversity are the result of rock record biases and correlate with metrics such as the numbers of formations, rock volume or outcrop area is the subject of much debate (Benton, 2015). There are three main hypothesised mechanisms for correlation: (*i*) a true bias, where diversity patterns are truly dependent on the rock record (Smith, 2001; Peters & Foote, 2001); (*ii*) common cause, where another factor such as sea level (and associated extent of shallow marine sea area and presence of epicontinental seas) drives correlations between the rock and fossil records (Peters, 2005, 2006; Peters & Heim, 2010, 2011; Hannisdal & Peters, 2011); and (*iii*) redundancy, where the effects of sampling on the fossil record and *vice versa* are redundant (Benton *et al*., 2011, 2013). Lloyd & Friedman (2013) reject the common cause hypothesis for fishes of Great Britain, but the mechanisms acting on the actinopterygian fossil record remain uncertain. The global actinopterygian fossil record includes both marine and freshwater components, which can be further divided into different zones [e.g. benthic assemblages (Sallan *et al*., 2018); open ocean *versus* shallow marine (Benson *et al*., 2010)] that may be subject to different drivers. For example, non‐marine area negatively correlates with diversity of shallow marine Mesozoic tetrapods, while contemporaneous open ocean diversity correlates with fossiliferous formations (Benson *et al*., 2010). The diversity of CEuropean marine mammals in the Cenozoic also does not correlate with rock outcrops (Marx, 2009). The actinopterygian record therefore represents an interesting test of the relative effects of these hypotheses. Analysis of actinopterygian richness in regions with adequate macrostratigraphic data (e.g. in North America; Peters, Husson & Czaplewski, 2018) may help to constrain the effect of geological biases acting on the Palaeozoic record. Richness in the Palaeozoic certainly correlates with geological proxies for sampling metrics (Figs 4 and S1), although the extent to which spatial bias impacts all of these metrics (including richness) is important to consider.

#### (b) Geographic and spatial biases

Europe and North America are the most intensely sampled regions in the marine animal fossil record (Close *et al*., 2020*b*). The vast majority of Palaeozoic actinopterygian occurrences are also from Europe and North America (Fig. 6), with important, although limited, occurrences from South America, Australia and Africa: this distribution is likely due to sampling intensity rather than true diversity. Sampling in the Devonian (Fig. 6A) and Carboniferous (Fig. 6B) is more restricted than the Permian (Fig. 6C), which may result from researcher biases towards the end‐Permian mass extinction and the general rise of terrestrial tetrapods. This same pattern is seen in terrestrial vertebrates of the same age (e.g. Dunne *et al*., 2018). Bias towards Europe and North America harks back to the early descriptions of actinopterygians (particularly from the UK), which are intimately linked to extensive mining, extraction and industrialisation of these regions during the 19th and early 20th centuries (e.g. Agassiz, 1833; King, 1850; Jackson, 1851). More broadly, recent work demonstrates just how important (neo‐)colonialism and global socio‐economics are as contributing factors to the global skew in palaeontological research outputs and therefore occurrence data (Raja *et al*., 2022). Variation in taxonomic practice can also impact richness counts depending on the number of researchers working on certain groups and time periods, and whether these researchers are the same for all time periods (Lloyd, Young & Smith, 2012*a*,*b*). This variation may contribute to higher diversity in Europe relative to other continental regions (Close *et al*., 2020*b*), although higher diversity is also likely intimately linked to historical factors and ongoing scientific colonialism (Raja *et al*., 2022).

Spatial biases also have a substantial impact on diversity trends at global scales due to temporal variability in the fossil content, fossil quantity, and palaeogeographical coverage of assemblages. The ‘global’ fossil record of any group in fact consists of occurrences distributed heterogeneously in space and time (Benson *et al*., 2016; Close *et al*., 2017, 2020*a*,*b*), and is better conceptualised as the sum of multiple regional records with different attributes (Close *et al*., 2020*a*). Diversity curves representing ‘global’ counts of taxa may therefore not be a true representation of the peaks and troughs in diversity of a group through time, but instead a combined record of the regional diversity in sampled areas. The effect of this is such that changes in diversity through time likely mainly mirror changes in the spatial extent of the group's fossil record between sampled intervals (Close *et al*., 2020*a*,*b*). Notably, the common cause (Peters, 2005, 2006; Peters & Heim, 2010, 2011; Hannisdal & Peters, 2011) and redundancy (Benton *et al*., 2011, 2013; Dunhill, Hannisdal & Benton, 2014; Benton, 2015) hypotheses do not explain this substantial source of sampling bias (Benson *et al*., 2016; Close *et al*., 2017, 2018, 2019, 2020*a*).

This is not to say that studies of the ‘global’ fossil record of specific taxonomic groups are uninformative, only that patterns must be carefully examined and interpreted with the knowledge that they likely exhibit significant spatial structuring. Diversity at the regional scale will be informative in determining specific drivers of, and biases in, the diversity signal (Crampton *et al*., 2003; Dunhill *et al*., 2012, 2013, 2014; Close *et al*., 2020*a*), as will examining differences between diversity measures (e.g. alpha and beta diversity), which can also be spatially dependent (Womack, Crampton & Hannah, 2021).

Different spatial biases acting on the freshwater and marine records may also variably impact different diversity estimates, dependent on the attributes of the sampled regions (Lagomarcino & Miller, 2012). For example, the species–area effect (Hallam & Wignall, 1999; Peters, 2005, 2007; Hannisdal & Peters, 2011; Close *et al*., 2020*b*) may play a role in levels of marine actinopterygian diversity, linked to changes in sea level and associated features (Lagomarcino & Miller, 2012; Jones *et al*., 2021), whereas other factors may drive freshwater actinopterygian diversity. Furthermore, the impacts of spatial and temporal variation in the establishment and reduction of epeiric seas (Peters, 2007) and reefs (Kiessling, Simpson & Foote, 2010) may play a role in determining diversity of actinopterygians through the Palaeozoic. These potential contributing factors would combine to result in complex drivers of regional heterogeneity in the actinopterygian fossil record, that can now be investigated with occurrence data.

#### (c) Taphonomic biases

Variation in the taphonomy of actinopterygian occurrences also likely influences interpretations of the Palaeozoic actinopterygian fossil record, but the impact of taphonomic processes and biases on this record has not been investigated. Taphonomic biases not only obscure underlying biological signals and impact perceived diversity, but likely influence understanding of other aspects of actinopterygian evolution, such as the degree of functional disparity or ecospace occupation (Smithwick & Stubbs, 2018). The effects of detrimental taphonomic processes varies geographically, between environments and with time (Brett, 1995; Zohar *et al*., 2008; Walker, Dunhill & Benton, 2020), although low‐energy, anoxic environments in which individuals were buried rapidly are usually those that best preserve vertebrates, i.e. Lagerstätten (Pardo, Lennie & Anderson, 2020). Lagerstätten play more of a role in biasing preservation in the marine record than the terrestrial (Muscente *et al*., 2017), and they clearly influence taxonomic diversity (Benson *et al*., 2010; Benson & Butler, 2011; Butler *et al*., 2011; Flannery Sutherland *et al*., 2019).

As with spatial biases, this may result in different preservational drivers of apparent diversity in the marine and non‐marine Palaeozoic; the majority of Lagerstätten yielding actinopterygians are indeed marine [e.g. Bear Gulch (Grogan & Lund, 2002; Lund *et al*., 2012); Glencartholm (Schram, 1983; Briggs & Gall, 1990); Gogo (Trinajstic, Briggs & Long, 2022); Kinney Brick Quarry (Lucas, DiMichele & Allen, 2021); Mazon Creek (Clements *et al*., 2019)], with comparatively few non‐marine sites (e.g. Montceau‐les‐Mines; Perrier & Charbonnier, 2014). Lagerstätten may skew diversity trends towards specific intervals, however they also provide unique snapshots of ecosystems in these intervals providing key information not only on taxonomic diversity, but also relative abundance within biota. For example, while actinopterygians were species‐poor relative to other vertebrates in the Devonian (Friedman, 2015), they are relatively abundant in the Gogo Formation (Trinajstic *et al*., 2022).

In recent years, literature has emerged on quantifying the skeletal completeness of the fossil record of various vertebrate groups using both character‐completeness metrics (e.g. Mannion & Upchurch, 2010; Brocklehurst & Fröbisch, 2014; Cashmore *et al*., 2020) and specimen‐based completeness metrics (e.g. Cleary *et al*., 2015; Tutin & Butler, 2017; Driscoll *et al*., 2019). To date, only one study has investigated completeness in a group of fishes (Schnetz *et al*., 2022), finding that the acanthodian fossil record is comprised predominantly of isolated remains and is among the least complete vertebrate records (measured as skeletal completeness). Completeness of specimens was significantly higher in freshwater deposits than marine, in contrast to the fossil record of marine tetrapod clades, which appears to be more complete than those of terrestrial tetrapods (Cleary *et al*., 2015; Tutin & Butler, 2017; Driscoll *et al*., 2019). Higher completeness in marine tetrapods is attributed to higher sedimentation rates in the marine realm, whereas anoxic conditions and low turbulence are suggested to be responsible for higher freshwater completeness in acanthodians (Schnetz *et al*., 2022). Quantification of the level of skeletal completeness in actinopterygians will help determine whether completeness of the actinopterygian record exhibits similar traits to the acanthodian or tetrapod records and aid interpretations of the biases acting on the fossil record, especially regarding marine *versus* freshwater fishes.

An additional taphonomic factor that may detrimentally impact our understanding of the actinopterygian fossil record is degree of preservation related to the size of specimens. There are data to suggest that larger organisms are much more likely to preserve than smaller organisms (Benson, 2018; Pardo *et al*., 2020), while more robust specimens can be associated with higher quality preservation (Cooper *et al*., 2006). The extent to which this applies to aquatic vertebrates is little understood, but this is likely to be of importance to actinopterygians: Sallan & Galimberti (2015) suggested that ray‐finned fish were small in the aftermath of the EDME. As the early Carboniferous coincides with the origin of the actinopterygian crown (Giles *et al*., 2017), and small ancestors are thought to have seeded most actinopterygian clades (Romano *et al*., 2016; Guinot & Cavin, 2018), a bias against preservation of smaller organisms may contribute to the failure to identify early members of these radiations. Furthermore, taphonomic factors have been shown to destroy small actinopterygian bones in particular (Smith, Stearley & Badgley, 1988) further confounding our ability to interpret the early actinopterygian fossil record correctly.

### (2) Taxonomic issues

Deep‐seated problems with Palaeozoic actinopterygian taxonomy exacerbate low levels of actinopterygian genus richness, despite high numbers of species and considerable morphological variation within these genera. Many genera from this period have apparently global distributions and stratigraphic ranges spanning nearly the entirety of the Carboniferous and Permian (Fig. 7; Gardiner, 1993; Sepkoski, 2002), which may be an artefact of reduced researcher effort in this period in favour of earlier Devonian forms, or later Mesozoic forms (Sallan, 2014). As a result, many mid‐late Palaeozoic actinopterygians have not been the subject of detailed taxonomic work.

Carboniferous and Permian actinopterygians received the most attention from researchers in the 19th and early 20th centuries. While much of this work was ground‐breaking and laid the foundations for palaeoichthyology, there are substantial problems with some outcomes of the research, notably the existence of wide‐ranging, poorly defined genera. Often, initial descriptions of taxa were brief and erected new genera with a heavy reliance on the shape of the body (e.g. deep‐bodied, fusiform, slender) and scale morphology (Agassiz, 1833; Traquair, 1877*a*, 1879; Moy‐Thomas & Dyne, 1938). This led to poorly defined genus diagnoses, often containing large numbers of dubiously related species – species whose characteristics sometimes even contradicted generic diagnoses. Some of the most notable problem genera – also termed ‘waste‐baskets’ (Evans, 2005) and ‘trash fish’ (Coates & Tietjen, 2018) – are ‘*Elonichthys*’ Giebel 1848 (44 species), *Rhadinichthys* Traquair 1877 (30 species), *Platysomus* Agassiz 1843 (20 species), *Amblypterus* Agassiz 1843 (18 species), *Palaeoniscum* Blainville 1818 (18 species) and *Acrolepis* Agassiz 1843 (14 species) (Mickle, 2017), although others exhibit similar issues (e.g. *Moythomasia* Gross 1950). Higher‐level taxonomic groups based on these genera, which are almost exclusively erected with generic diagnoses (Sallan, 2014), suffer from the same problems.

In addition to being taxonomically ambiguous, these few Palaeozoic actinopterygian genera likely obscure a significant proportion of genus‐level diversity. Redescriptions and redefined diagnoses are necessary in order to reveal the true taxonomic diversity hiding within these genera. Recently, *Elonichthys* was redefined to include just three species (Schindler, 2018*a*) from Central European late Carboniferous and early Permian deposits. Consequently, the temporal and spatial extent of the genus has been drastically reduced, and around 50 other nominal species of ‘*Elonichthys*’, ranging from the Tournaisian to the Wuchiapingian, are invalid and currently unaccounted for in genus‐level diversity analysis. More broadly, while apparently widespread by modern continental configuration (Fig. 6), palaeogeographic distributions of problem genera are more concentrated due to the proximity of Western Europe and North America in the Palaeozoic (Scotese, 2021). That these regions, where the majority of species within problem genera occur (Fig. 7), were geographically contiguous in the Palaeozoic exacerbates the geographic research bias associated with greater sampling of North America and Europe. Shared presence of numerous groups of actinopterygians [e.g. haplolepids, eurynotiforms, aeduelliforms (Sallan & Coates, 2013; Elliott, 2014, 2016; Hodnett & Lucas, 2015)] in present‐day North America and Western Europe provides further evidence of a close link.

In recent years, new anatomical information revealed by CT scanning has prompted several reinvestigations of the validity of Palaeozoic taxa. Coates & Tietjen (2018) recently redescribed a Bashkirian actinopterygian and moved it to *Trawdenia* n. gen. This specimen was originally referred to *Mesopoma*, a taxon erected by Traquair (1890) in an attempt to separate species belonging to *Canobius* and *Rhadinichthys*. Traquair subsequently retracted the genus (Traquair, 1912), before Moy‐Thomas & Dyne (1938) restored it (see Coates, 1993, 1998; Coates & Tietjen, 2018). *Trawdenia* exemplifies both the root cause of the problem with many Carboniferous and Permian actinopterygian genera – a diagnosis based on characteristics prevalent in other late Palaeozoic actinopterygians and lacking unambiguous synapomorphies – and also the route to resolving the problem: detailed redescription to identify unique characters aided by currently available technology such as CT scanning. Reinvestigation of Palaeozoic material is not simply an exercise in correcting taxonomy, however. Coates (1999) and Coates & Tietjen's (2018) work revealed previously hidden features of the endocast and pectoral fin in a specimen that had been known to the literature for over a century. The case of *Trawdenia*, as well as others such as *Eurynotus crenatus* (Friedman *et al*., 2018) and *Brazilichthys macrognathus* (Figueroa *et al*., 2019), clearly demonstrate that reinvestigation can reveal not only hidden taxa, but untold anatomical and ecological diversity.

### (3) Phylogenetic issues

Relationships of the four extant actinopterygian clades (Cladistia, Chondrostei, Holostei, Teleostei) has reached a point of consensus through both molecular (e.g. Betancur‐R *et al*., 2017; Hughes *et al*., 2018; Dornburg & Near, 2021) and morphological (e.g. Patterson, 1982; Gardiner & Schaeffer, 1989; Coates, 1998; Cloutier & Arratia, 2004; Grande, 2010; Xu, Gao & Finarelli, 2014; Giles *et al*., 2017) research. Sallan (2014) provided a detailed summary of previous hypotheses of living clades and the basis for this consensus. Friedman (2015) synthesises attempts to place Palaeozoic actinopterygians relative to extant clades, highlighting that the relationships of extinct actinopterygians, both in relation to each other and to extant clades, remain unclear.

**Fig. 9:**
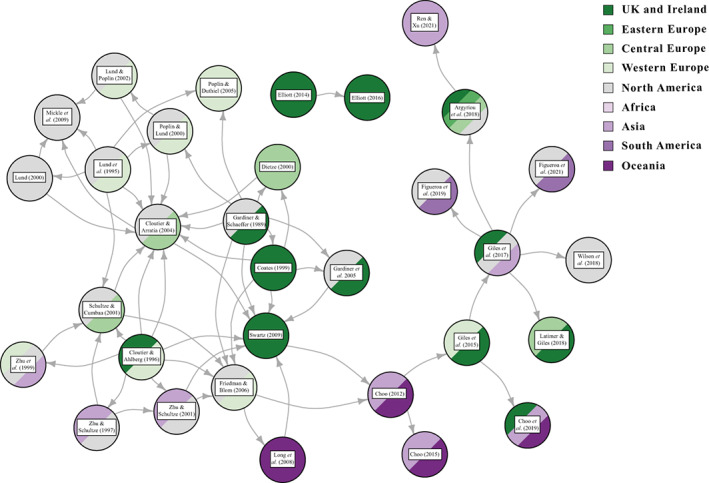
Network depicting the flow of characters to and from phylogenetic analyses of Palaeozoic actinopterygians. Nodes are coloured according to the geographic region in which authors' listed institutions (in the primary article) are located.

The cladistic analysis of Gardiner & Schaeffer (1989), which built significantly on prior work by Gardiner (1984), represented a seminal study for investigations into Palaeozoic actinopterygian relationships (Friedman, 2015). Gardiner & Schaeffer (1989) organised early actinopterygians into groups (e.g. the *Moythomasia* Group, the *Platysomus* Group), which they tentatively posited to be monophyletic, in order to determine actinopterygian phylogeny. Although the monophyly of these groups was rarely upheld in later work, this analysis and the anatomical characters it established forms the basis for almost all future phylogenetic studies (e.g. Coates, 1999; Dietze, 2000; Poplin & Lund, 2000; Cloutier & Arratia, 2004; Poplin & Dutheil, 2005; Friedman & Blom, 2006; Swartz, 2009; Fig. 9). The history of phylogenetic work on actinopterygians mirrors the geographic biases related to sampling of actinopterygian occurrences, with clear bias towards the regions in which research groups are located (Fig. 9).

Subsequent analyses have attempted to determine the relationships of primitive actinopterygians relative to extant clades (e.g. Cloutier & Arratia, 2004) or focussed solely on Palaeozoic actinopterygian interrelationships (e.g. Friedman & Blom, 2006). Although most analyses draw on multiple sources, two main subsequent ‘lineages’ of analyses have arisen, both with a focus shifted towards relationships of actinopterygians rather than early bony fishes. Cloutier & Arratia (2004), which attempted a major synthesis of existing character matrices, sourcing characters from previous cladistic and phylogenetic studies, heavily influenced Mickle *et al*. (2009) and Swartz (2009), while Friedman & Blom (2006) became the basis of Choo (2012) and all subsequent analyses derived from that matrix (Giles *et al*., 2015, 2017; Argyriou *et al*., 2018; Choo *et al*., 2019; Figueroa *et al*., 2019, 2021; Fig. 9). Giles *et al*. (2017) significantly expanded and revised this derived matrix with the aim of interrogating relationships between living and fossil actinopterygian lineages.

**Fig. 10:**
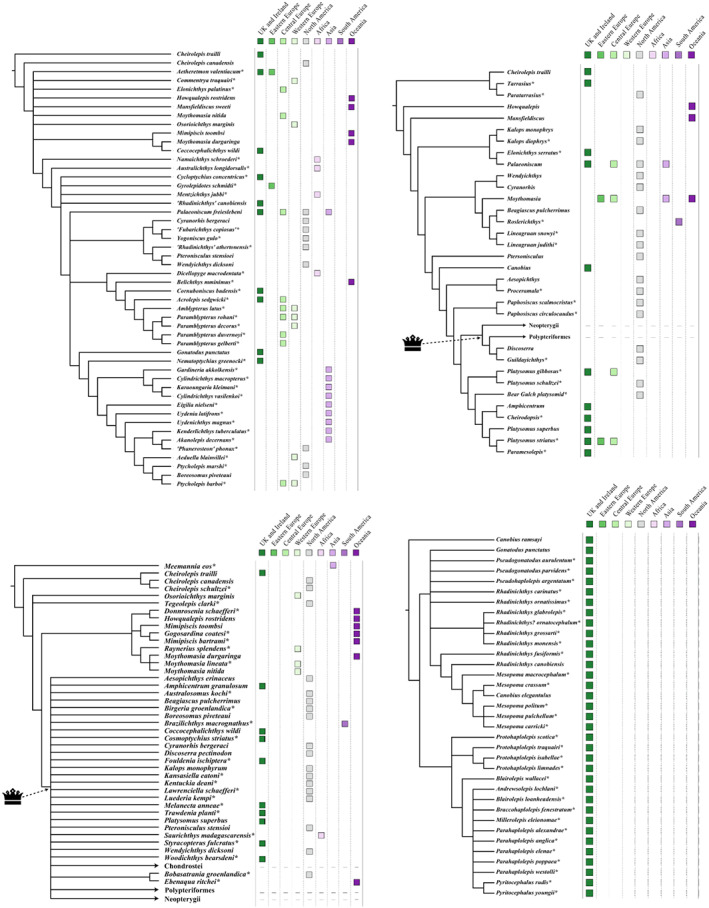
Phylogenetic analyses of Palaeozoic actinopterygian relationships showing the geographic distribution of sampled taxa: (A) Dietze (2000); (B) Mickle *et al*. (2009); (C) Figueroa *et al*. (2019); and (D) Elliott (2016). The actinopterygian crown node is indicated in analyses that include extant taxa. Asterisks (*) indicate taxa not included in the other depicted analyses to show the lack of overlap between datasets.

Other matrices focus on a particular fauna or geographic region. Most notable amongst these are efforts to investigate the relationships of Bear Gulch actinopterygians, which include limited taxa from outside this deposit (Lund, Poplin & McCarthy, 1995; Lund, 2000; Fig. 9). Cloutier & Arratia (2004) attempted to integrate these analyses with other early actinopterygian and osteichthyan matrices. This was further expanded by Mickle *et al*. (2009; Fig. 10B; and in an unpublished thesis: Mickle, 2012), who included more Bear Gulch forms and several other taxa (e.g. *Roslerichthys*; Hamel, 2005). Separately, Elliott (2016; Fig. 10D) conducted an analysis of Scottish Bashkirian actinopterygians, sampling traditionally underrepresented groups (such as haplolepids) while excluding all other Palaeozoic actinopterygians.

In general, expansion of these matrices has focused on increasing taxon sampling of actinopterygian groups that are already represented and adding more crownward taxa (Latimer & Giles, 2018; Argyriou *et al*., 2018; Ren & Xu, 2021), rather than including previously excluded Palaeozoic taxa. Numerous proposed Palaeozoic actinopterygian clades are yet to be included in phylogenetic analyses despite high support in the literature [e.g. eurynotiforms (Sallan & Coates, 2013; Friedman *et al*., 2018)], and many others remain represented by a single terminal (e.g. platysomids; Giles *et al*., 2017). Hypotheses of relationships have been shown to change substantially when additional taxa from underrepresented taxonomic groups are included [e.g. saurichthyids (Argyriou *et al*., 2018); dapediids and pycnodonts (Latimer & Giles, 2018)], resulting in topologies and divergence timelines inconsistent with past molecular (Betancur‐R *et al*., 2017; Hughes *et al*., 2018; Dornburg & Near, 2021) and morphological (Cloutier & Arratia, 2004; Grande, 2010; Xu *et al*., 2014; Giles *et al*., 2017) consensus, suggesting that this skewed representation may be a major source of uncertainty in the early actinopterygian tree.

Other sources of bias are noticeable, especially temporal and geographic imbalances. Most analyses contain roughly even numbers of Devonian and Carboniferous taxa (Coates, 1999; Cloutier & Arratia, 2004; Gardiner, Schaeffer & Masserie, 2005; Giles *et al*., 2017; Figueroa *et al*., 2021), despite there being an order of magnitude more species described from the Carboniferous (Fig. 4). Permian taxa are largely excluded, despite the nearly equivalent numbers of Permian species relative to the Carboniferous (Fig. 4). Furthermore, most analyses heavily sample fishes from British, North American and Australian deposits (Fig. 10). Dietze (2000; Fig. 10A) is a notable exception that incorporates underrepresented taxonomic groups (e.g. amplypterids) and geographic regions (e.g. Africa, Asia, Central Europe), although very few subsequent analyses have built upon this widespread sampling of taxa. A synthesis and integration of disparate phylogenetic analyses that focus on individual groups or regions (e.g. Dietze, 2000; Elliott, 2016) with broader analyses that attempt to span the actinopterygian radiation (e.g. Giles *et al*., 2017; Figueroa *et al*., 2019, 2021; Ren & Xu, 2021) is sorely needed. Among the most recent iterations of the Giles *et al*. (2017) matrix are studies beginning to expand the geographic spread of taxa by including actinopterygians from South America (Figueroa *et al*., 2019, 2021). Importantly, when sampling expands beyond taxa from the Euro‐American realm, support for past hypotheses of relationships among stem‐actinopterygians collapses (Fig. 10C).

Lack of an adequate representation of known morphologies, clades and geographic regions in character matrices is drastically preventing an accurate understanding of Palaeozoic actinopterygians (Friedman, 2015). While revisions to early cladistic and phylogenetic analyses (Gardiner & Schaeffer, 1989; Coates, 1999) have resulted in the shift of the majority of Palaeozoic taxa from the actinopterygian crown to the stem (Cloutier & Arratia, 2004; Mickle *et al*., 2009; Giles *et al*. 2017), the relationships between the numerous diverse clades within the Palaeozoic are still highly unstable. As well as expanding the geographic range encompassed by taxa, it will be important to address existing imbalances in geographic sampling before analysing important phylogeographic aspects of actinopterygian evolution (such as dispersal rates), as variation in sampling can greatly influence results (Gardner, Surya & Organ, 2019).

## VI. CONCLUSIONS

(1) Comprehensive occurrence‐based datasets are necessary for examining biases in the fossil record and deducing accurate diversity trends (Alroy, 2020), while robust phylogenies are crucial for detailed macroevolutionary analyses (Soul & Wright, 2021). Fishes are rarely the subject of such analyses, but present ample opportunities for investigating evolutionary dynamics through the Palaeozoic. A priority for Palaeozoic actinopterygian research is to record occurrences in the PBDB and update regional substage ages; efforts to do so are in progress.
(2) Actinopterygian richness fluctuates throughout the Palaeozoic, but raw counts largely appear to be tracking sampling proxies. Europe and North America are oversampled in comparison to most Global South regions, and sampling and spatial biases have a clear influence on the record. Targeted sampling of underrepresented regions (e.g. mid‐ to high‐palaeolatitudes in the Carboniferous), time periods and environments (e.g. marine environments in the Late Carboniferous–Middle Permian) will be necessary to redress this imbalance. Concurrently, detailed sampling of well‐known regions will allow for more accurate diversity analyses of local and regional subsets, which are a critical avenue for research considering issues regarding analysis of ‘global’ fossil records. This detailed sampling should take the form of cataloguing museum collections in such regions (as well as more broadly) given the likelihood that they contain a large number of occurrences that are not recorded in the primary literature, and thus not entered into occurrence databases (Marshall *et al*., 2018).
(3) Our occurrence‐level database for Palaeozoic actinopterygians paves the way for examining biases in the fossil record and deducing accurate diversity trends. In particular, analytical methods of sampling standardisation (Chao, 1984; Chao & Jost, 2012; Alroy, 2017, 2018, 2020; Close *et al*., 2020*a*; Jones *et al*., 2021) and application of diversity estimation at different scales (Close *et al*., 2019) represent a priority for future studies (Alroy, 2010*a*,*b*; Close *et al*., 2018). Incorporation of macrostratigraphic data may help facilitate a synthesis of the various biases impacting the actinopterygian fossil record.
(4) Historical poor taxonomic practices mask valuable taxonomic and morphological diversity in Palaeozoic actinopterygians (Coates & Tietjen, 2018; Schindler, 2018*a*). Re‐evaluation of ‘waste‐basket’ taxa, aided by CT scanning, represents the foundation for many other studies. Taxonomic revisions will result in a tightening of the geographic and temporal ranges of these widespread genera, which in turn will help to deduce accurate patterns of palaeodiversity (Close *et al*., 2018), palaeogeographic dispersal (Cavin, 2008; Gardner *et al*., 2019) and regional connectedness (Stigall *et al*., 2017). Redescriptions also aid the identification of early members of extant actinopterygian clades (Giles *et al*., 2017), thus paving the way for a better understanding of the evolutionary dynamics between clades (Clarke, Lloyd & Friedman, 2016) as actinopterygians became dominant in aquatic habitats (Sallan & Coates, 2010; Friedman, 2015).
(5) Existing phylogenetic character matrices are plagued by similar biases to the overall fossil record, heavily oversampling North American and European fishes, and expanding the geographic and temporal range of phylogenies must represent a priority. Continued addition of taxa and well‐formulated characters (Brazeau, 2011), as well as better methods for dealing with inapplicable characters (Brazeau, Guillerme & Smith, 2019; Goloboff *et al*., 2021), will generate robust hypotheses of relationships with which to investigate key evolutionary events. Greater incorporation of techniques such as tip‐dating may be able to tease apart relationships suspected to result from homoplasy (Lee & Yates, 2018), for example the multiple deep‐bodied radiations of Palaeozoic actinopterygians.
(6) The mechanisms underlying actinopterygian diversification following the end‐Devonian mass extinction and their subsequent evolutionary dynamics through the Palaeozoic remain largely unknown, in part due to the lack of stable phylogenetic hypotheses of relationships and occurrence databases. Having these data in hand will enable a wide range of analysis, from inference‐based methods to phylogenetic comparative methods to palaeogeographical dispersal:
  (a) Reliable and representative phylogenies are an important component of biogeographic network analyses (Button *et al*., 2017; Dunne *et al*., 2018; Kubo, 2019), and alternative estimates of diversity such as lineages counts through time (also referred to as phylogenetic diversity; Ezcurra & Butler, 2018) that would complement taxic estimates of diversity.
  (b) Application of phylogenetic comparative methods has the potential to identify adaptive radiations (Close *et al*., 2015; Ezcurra & Butler, 2018; Felice & Goswami, 2018; Halliday *et al*., 2019; Simões *et al*., 2020). Actinopterygians appear to diversify explosively in the early Carboniferous, but the lack of comprehensive phylogenetic analysis prevents testing of whether this best fits a model of classic extinction recovery, adaptive radiation, or ecological release (Schluter, 2000; Sallan & Friedman, 2012; Friedman & Sallan, 2012; Slater, 2013). In tandem, investigating survivorship and selectivity through mass extinctions, such as the end‐Devonian, among and between lineages (Soul & Friedman, 2017; Allen *et al*., 2019) may reveal more detail on the effects of mass extinctions (Sallan & Friedman, 2012; Sallan & Galimberti, 2015). Deep‐bodied Palaeozoic actinopterygians also represent an obvious test case for exploring these techniques, for example by quantifying convergence and teasing this apart from shared history (Speed & Arbuckle, 2017; Arbour & Zanno, 2020).
  (c) Previous work has examined shifts between marine and non‐marine habitats in other fossil groups (and coincident changes in morphology and disparity; Lamsdell, 2016). By combining palaeoecological observations from occurrence data with reliable phylogenetic hypotheses, it will be possible to examine habitat transitions and trends in actinopterygian ecology and biogeography through time (Lamsdell *et al*., 2017). In addition, previous ancestral‐state based hypotheses of crown‐group actinopterygian habitats have inferred both a freshwater (Carrete Vega & Wiens, 2012) and marine (Betancur‐R, Ortí & Pyron, 2015; Guinot & Cavin, 2018) origin for actinopterygians (although the result indicates that a freshwater origin is due to the absence of fossil data in the analysis). Given recent upheavals in established schemes of phylogenetic relationships, with a particular effect on deep‐branching members of stem groups (e.g. Giles *et al*., 2017), ancestral state reconstructions should be reassessed. As it may be physiologically easier to adapt from one environment to another (Betancur‐R *et al*., 2015), it would be prudent to explore the use of asymmetric transition models as recently used to investigate the evolution of oviparity and viviparity in squamates (Blackburn, 2015).

Collectively, these investigations will greatly expand our understanding of the early evolution and rise to dominance of the most speciose extant vertebrate clade, the Actinopterygii.

## Supporting information


**Fig. S1.** Regressions of total genus richness in individual equal‐length stages with (A) number of localities, (B) number of geological formations, (C) number of occupied equal‐area grid cells, (D) stage length, and (E) sea level; and regressions of freshwater genus richness (F) and marine genus richness (G) with sea level, including Devonian stages, and of overall genus richness (H) and freshwater genus richness (I) with sea level, excluding Devonian stages.

**Table S1.** Occurrences of Palaeozoic actinopterygians (ma, million years ago; myr, million years).

**Table S2.** Description of the roughly equal‐length time intervals into which occurrence data were placed (ma, million years ago; myr, million years).
