# Supplementary material for: The early diversification of ray‐finned fishes (Actinopterygii): hypotheses, challenges and future prospects
Source: Biol Rev Camb Philos Soc. 2022 Oct 3;98(1):284–315. doi: 10.1111/brv.12907 (PMC10091770; doi:10.1111/brv.12907)
Supplement: Supplementary file 1 — Fig. S1. Regressions of total genus richness in individual equal‐length stages with (A) number of localities, (B) number of geological formations, (C) number of occupied equal‐area grid cells, (D) stage length, and (E) sea level; and regressions of freshwater genus richness (F) and marine genus richness (G) with sea level, including Devonian stages, and of overall genus richness (H) and freshwater genus richness (I) with sea level, excluding Devonian stages. [file BRV-98-284-s001.pdf]

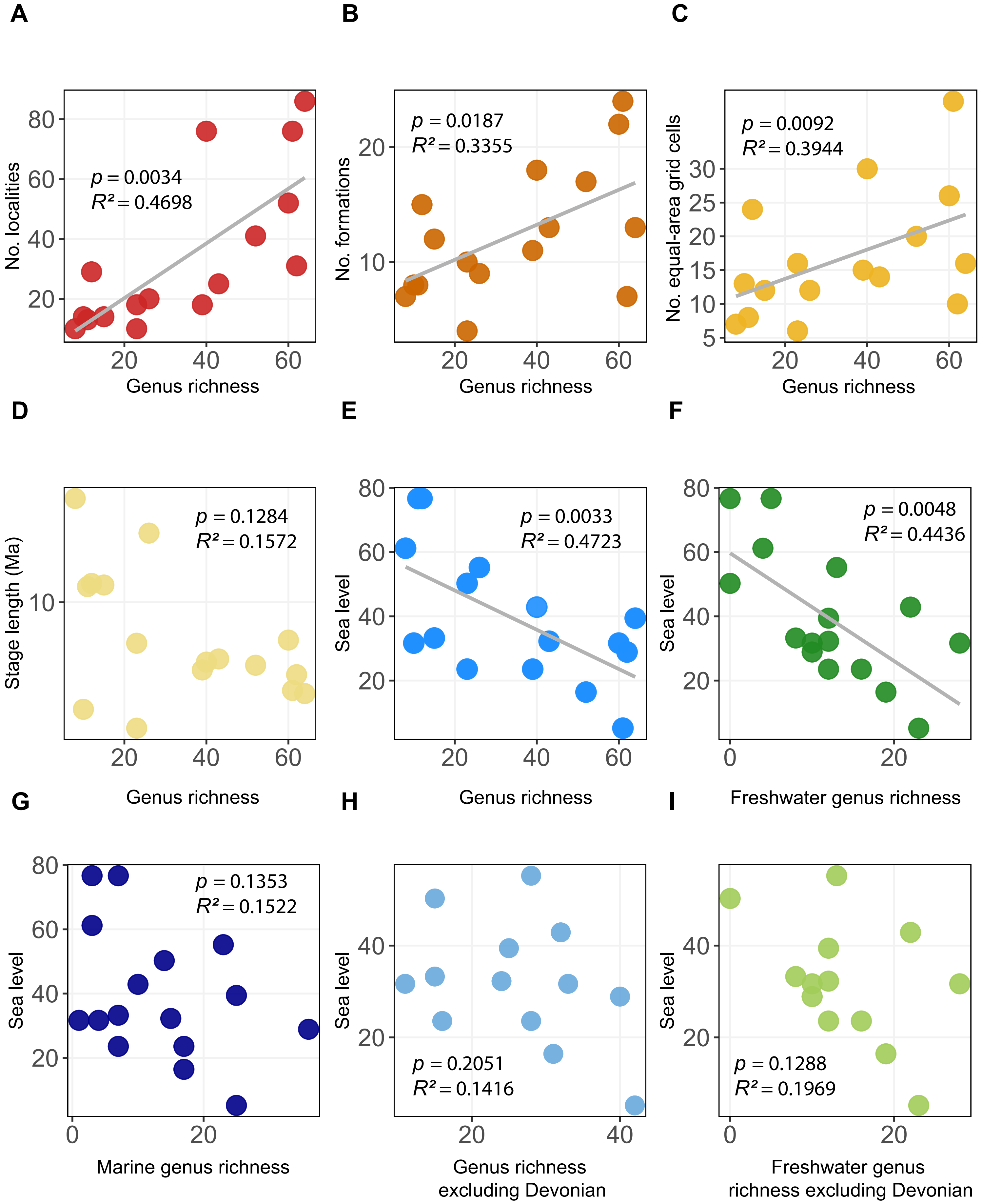

Fig. S1. Regressions of total genus richness in individual equal-length stages with (A) number of localities, (B) number of geological formations, (C) number of occupied equal-area grid cells, (D) stage length, and (E) sea level; and regressions of freshwater genus richness (F) and marine genus richness (G) with sea level, including Devonian stages, and of overall genus richness (H) and freshwater genus richness (I) with sea level, excluding Devonian stages.
